# Upregulation of innate and adaptive immune mechanisms facilitating prevention of gastric *Helicobacter pylori* infection in guinea pigs by *per os* administration of chitosan microparticles loaded with *Mycobacterium bovis* BCG

**DOI:** 10.3389/fimmu.2026.1771052

**Published:** 2026-03-18

**Authors:** Weronika Gonciarz, Marek Brzeziński, Agnieszka Wosiak, Agnieszka Jeleń, Ewa Balcerczak, Magdalena Chmiela

**Affiliations:** 1 Department of Immunology and Infectious Biology, Institute of Microbiology, Biotechnology and Immunology, Faculty of Biology and Environmental Protection, University of Lodz, Lodz, Poland; 2 Centre of Molecular and Macromolecular Studies, Polish Academy of Sciences, Lodz, Poland; 3 Department of Pharmaceutical Biochemistry and Molecular Diagnostics, Medical University of Lodz, Lodz, Poland; 4 Laboratory of Molecular Diagnostics, BRaIn Laboratories, Medical University of Lodz, Lodz, Poland

**Keywords:** bone marrow-derived macrophages, chitosan microparticles, *Helicobacter pylori*, immune response, *Mycobacterium bovis* BCG

## Abstract

**Background:**

*Helicobacter pylori* (*H. pylori*) rods frequently colonize and damage the gastric mucosa in humans, causing inflammation, gastric or duodenal ulcers and even gastric cancer. *H. pylori* negatively modulates the activity of immune cells, including macrophages and lymphocytes facilitating the persistence of infection. Increasing resistance of *H. pylori* isolates to commonly used antibiotics diminishes the success of therapy. These prompt searches for new therapeutic formulations to improve the effectiveness of immune mechanisms against *H. pylori*. Based on the previous *in vitro* studies indicating the immunomodulatory properties of *Mycobacterium bovis-*(Bacillus Calmette–Guérin) BCG vaccine bacilli, we developed chitosan microparticles-(CHI MPs) modified with N-acetylglucosamine-(G) or with Pluronic F-127-(P) to facilitate the delivery and persistence of live *M. bovis* BCG in the stomach and in the gut of *Cavia porcellus* susceptible to *H. pylori* infection.

**Methods:**

Animals (5/per group) were inoculated *per os* only with CHI MPs, G or P variant or both, or with such CHI MPs and then with the reference *H. pylori* CCUG17874 positive for cytotoxin-associated gene A-(*cagA*) (3 times in two-day intervals). Control animals received only *H. pylori* (positive control) or *Brucella* broth (negative control). Two assessment points have been selected: 7 and 28 days after the last *H. pylori* inoculation, mimicking early and chronic infection, respectively.

**Results:**

The gastric tissue of guinea pigs (4/5) receiving G/P CHI MPs loaded with *M. bovis* BCG before inoculation with *H. pylori* was not colonized with these bacteria after 28 days as shown by quantitative *cagA* polymerase chain reaction. Protection in this group was associated with an increased number of myeloid precursors in the bone marrow and enhanced macrophage infiltration in the gastric tissue. The bone marrow-derived macrophages from this group showed enhanced phagocytic activity, whereas in animals inoculated only with *H. pylori*, this activity was negatively modulated. The protective effect of the studied CHI MPs was also associated with increased gastric concentrations of secretory IgA and enhanced splenocyte proliferation.

**Conclusions:**

The obtained results indicate the immunomodulatory potential of CHI MPs loaded with *M. bovis* BCG to improve innate and adaptive immune mechanisms, facilitating control of *H. pylori* infection in the guinea pig model.

## 1 Introduction

*Helicobacter pylori*, spiral Gram-negative rods described for the first time by Warren and Marshal in 1983, colonize the gastric mucosa in approximately 50% of the human population (1). They trigger chronic inflammatory responses, gastric or duodenal ulcers and gastric cancer or colorectal cancer (1–3). These bacteria can also contribute in the development of some extra-gastric diseases (4–14). In the stomach, *H. pylori* disrupts the gastric epithelial barrier by increasing oxidative stress and cell apoptosis (15–17). The World Health Organization (WHO) reports indicate that resistance of *H. pylori* to antibiotics such as amoxicillin, clarithromycin, metronidazole, and levofloxacin, which are used to treat infections, is permanently increasing (18–23), coinciding with evading by *H. pylori* host immune responses, which may impact the effectiveness of eradication efforts. Components of *H. pylori* inhibit phagocytosis, reduce the cytotoxic activity of natural killer (NK) cells (24–29) and lymphocyte proliferation (30, 31). Some *H. pylori* components share sequences with host proteins, potentially triggering the formation of autoreactive antibodies (6).

Inhibition of immunocompetent cell activity, particularly macrophages by *H. pylori* components prompts the search for biological preparations with immunomodulatory potential to improve the host’s antibacterial immunity. Macrophages utilize phagocytosis to eliminate infectious agents and dead cells, thereby helping to restore local homeostasis (32, 33).

The *Mycobacterium bovis* BCG vaccine strain is regarded as a potential immunomodulator of macrophage activity via mechanisms termed “immune training”, which induce non-specific immune memory in innate immune cells, potentially offering cross-protection against unrelated pathogens (34–36). Several studies have revealed that BCG vaccination may induce a better innate immune response against *Candida albicans, Staphylococcus aureus*, respiratory syncytial virus (RSV), and potentially severe acute respiratory syndrome virus (SARS-CoV-2) (37, 38). The *M. bovis* onco-BCG formula has been successfully used for immunotherapy in bladder cancer (39). BCG mycobacteria induce oxidative stress and apoptosis in tumor cells (39), activate immunocompetent cells that eliminate tumor cells (40). In a previous *in vitro* study, we showed that *M. bovis* BCG strain helped restore the phagocytic activity of human THP-1-derived macrophages that had been diminished by *H. pylori* components (41).

An experimental model of *H. pylori* infection in guinea pigs was previously characterized by us in terms of inflammatory and immune responses initiated by these bacteria and the induction of regeneration processes (15, 31, 42).

In this study, we used this model to determine whether *per os* inoculation of animals with chitosan microparticles (CHI MPs) containing live *M. bovis* BCG, which are released in the stomach or in the gut, or simultaneously in both localizations, can result in upregulation of innate and adaptive immune responses facilitating *H. pylori* elimination in conjunction with initiation of gastric tissue regeneration. Animals were inoculated *per os* with *H. pylori* alone, CHI MPs loaded with *M. bovis*-BCG modified with N-acetylglucosamine (CHI MPs BCG (G)) to enhance the adhesion of CHI MPs to gastric mucin or with CHI MPs modified with Pluronic F127 and loaded with *M. bovis*-BCG (CHI MPs BCG (P)), to facilitate the release of the bacterial cargo in the gut. Such CHI MPs were administered to animals separately or together, or animals received CHI MPs first and were then infected with *H. pylori*.

Given the diminishing responsiveness of macrophages to *H. pylori* components (24, 26), and the role of *M. bovis* BCG in the immune training of innate immune cells (41), we examined macrophage infiltration in the gastric tissue of the studied animals. We assessed the phenotype of bone marrow cells based on selected surface markers of myeloid precursors (CD34/CD117/Ly6G), and the secretion of pro-inflammatory (TNF-α, IL-8) and anti-inflammatory/regulatory (IL-10) cytokines by bone marrow-derived macrophages (BMDM).

Using BMDM from animals inoculated with different variants of CHI MPs loaded with *M. bovis* BCG we determined the phagocytic capacity of these cells and expression of CD11d integrin, which drives macrophage migration and extravasation and is involved in phagocytosis. Considering the concept of immune training of macrophages by *M. bovis* BCG, in BMDM the histone 3 lysine 4 (H3K4) methyltransferase methylation was assessed.

As innate and adaptive immune responses cooperate, we determined the production of secretory (sIgA) antibodies in the gastric tissue of studied animals and the number of lymphatic follicles with germinal centers (GC) in the spleen, in association with proliferation of spleen lymphocytes *ex vivo* in response to *H. pylori* antigens or *M. bovis* BCG in comparison with spontaneous reaction of spleen lymphocytes or induced by phytohemagglutinin (PHA) as positive control.

## 2 Materials and methods

### 2.1 Chitosan microparticles loaded with *M. bovis* BCG

The studied CHI MPs were produced by spray drying using a mini spray dryer (B-290, Buchi, Switzerland) as previously described (43), and according the patent application with a number P.447595 (“Method for obtaining a biopolymer carrier of *Mycobacterium bovis* BCG vaccine bacilli for combating *Helicobacter pylori* infection”. Three distinct solutions were prepared in 1% (v/v) acetic acid (1): chitosan (product number: 448877Merck Millipore, Burlington, Massachusetts, USA; medium molecular weight (MMW) 190–310 kDa, 75–85% deacetylated, viscosity: 200–800 cP, 1 wt. % in 1% acetic acid (25°C, Brookfield) (lit.); InChI Key: FLASNYPZGWUPSU-SICDJOISSA-N) (2), chitosan with N-acetylglucosamine GalNAc (G) (Merck Millipore, Burlington, Massachusetts, USA), and (3) chitosan combined with Pluronic F127 (P) (Merck Millipore, Burlington, Massachusetts, USA). We added 1 mL of *M. bovis* BCG (3×10^8^ colony-forming units [CFU]/mL) (Synthaverse, Lublin, Poland), to each solution to generate *M. bovis* BCG-loaded microparticles. Ultimately, the particles were collected from the cyclone and placed in a glass vial. The obtained CHI MPs have been characterized, including the size distribution, zeta potential, loading efficiency, the viability of *M. bovis* BCG and the kinetics of release of mycobacteria in the pH=3.0, pH=7.2, and pH=8.8 over time as previously described (43). After spray-drying, the viability of *M. bovis* BCG bacilli in chitosan microparticles was qualitatively assessed by fluorescence microscopy using BacLight™ dye or microbiologically by the growth of bacteria on Löwenstein-Jensen medium (43).

### 2.2 *Helicobacter pylori* culture

For inoculating guinea pigs, we used *H. pylori* reference strain 17874 from CCUG (Culture Collection University of Gothenburg, Sweden), which was positive for the vacuolating cytotoxin (VacA) and cytotoxin associated gene A (CagA) protein. *H. pylori* strains were preserved at –80°C in Tris-buffered saline (TBS) with 20% glycerol and 10% heat-inactivated fetal calf serum (FCS). The bacteria were cultured for 5 days on modified Helicobacter agar (Becton, Dickinson, Heidelberg, Germany) under microaerophilic conditions (using GasPak, Becton, Dickinson, Heidelberg, Germany) at 37°C. Every 48 hours, the bacteria were transferred to fresh Helicobacter agar and cultured as above. At each transfer, urease, catalase, and oxidase production were tested, and Gram staining was performed to eliminate coccoidal bacterial forms.

### 2.3 Guinea pig model

All experiments involving animals were developed according to the Animal Research: Reporting of *In Vivo* Experiments (ARRIVE) guidelines and guidelines and regulations European Union (EU) directive (Directive 2010/63/EU of the European Parliament and of the Council of 22 September 2010 on the protection of animals used for scientific purposes (Dz.U. L 276 z 20.10.2010, s. 33–79), and were approved by the Local Ethics Committee (LKE9) for Animal Experiments of the Medical University of Lodz, Poland, which was established by the Ministry of Science and Higher Education in Poland (Ethics Committee decision number: ŁB 41/277/2023).

The 75 three-month-old Himalayan guinea pigs (*Cavia porcellus*) were free of pathogens (five males and five females per group), and housed in the Animal Facility at the Faculty of Biology and Environmental Protection, University of Lodz (Poland), kept in cages with free access to drinking water, and fed standard chow.

The animals received 1 mL *per os* of 0.2 N NaHCO_3_ (without anesthesia) for neutralization of acidity of the stomach juice, and then after 10 min received 1 mL (without anesthesia) of studied suspensions: only suspension of the reference *H. pylori* strain CCUG 17874 (1×10^10^ CFU/mL) three times in two-day intervals (n=5); only CHI MPs loaded with *M. bovis* BCG (1×10^8^ CFU/mL): CHI BCG (P) (n=5), or CHI GlcNAc BCG (G) (n=5), or CHI BCG (P) and CHI GlcNAc BCG (G) simultaneously, two times in seven-day intervals (n=5), or firstly such CHI MPs two times in seven-day intervals and then *H. pylori* CCUG 17874 (1×10^8^ CFU/mL) three times in two-day intervals (n=5). Control animals received only Brucella broth three times in two-day intervals (n=5). CHI MPs loaded with *M. bovis* BCG were administered twice at 7-day intervals to induce immune training: day 1 - the first dose of CHI MPs loaded with *M. bovis* BCG (immune training), 6 days of rest, day 7 - the second dose of CHI MPs loaded with *M. bovis* BCG (restimulation of immune cells).

Animals were monitored daily for body weight, water and food intake, behavioral symptoms, skin and fur condition, body temperature, heart rate, respiration, and diarrhea. No abnormal behavioral or physiological changes were observed in animals under the experiment. 7 and 28 days after the last inoculation, animals were euthanized by an overdose of sodium barbiturates (Morbital, Biowest, Puławy, Poland). Blood was collected from all 75 animals to obtain serum, and the stomach, spleen, and femur to obtain tissues or to isolate bone marrow macrophages, respectively for further investigation.

### 2.4 *Helicobacter pylori* status

The *H. pylori* infection in guinea pigs was confirmed by the detection of Helicobacter-like organisms (HLO) in thin-layer sections of stomach tissue from the pyloric region after standardized Giemsa staining and by imaging of gastric tissue by scanning electron microscopy (SEM) or based on the characteristic infrared (IR) spectrum of the serum sample (44) as well as by quantitative molecular assay – RT-qPCR to assess the *cagA* copy number.

#### 2.4.1 Giemsa staining

Guinea pig tissue was fixed in 10% formaldehyde and embedded in paraffin. Thick sections (3 μm) were stained using a routine histological procedure with the Giemsa stain solution and analyzed in a light microscope for the detection of HLO based on selected criteria of histological analysis of tissue sections called the Sydney system, as previously described (42, 45). The test material was coded and assessed twice by two independent persons.

#### 2.4.2 SEM analysis of gastric tissue

The morphology of gastric tissue from *H. pylori-*infected animals, or from control uninfected animals, was examined using SEM, as described by Carron et al. (2006) (46). Briefly, a section of the guinea pig stomach antrum was washed and then dried in a sterile container for 3 hours at 60°C. The control was the reference strain *H. pylori*, cultured as described above. Next, before imaging, the samples were coated with a thin layer of gold (about 20 nm) and analyzed with a Jeol JSM-6010LA (JEOL Ltd., Japan). The microscope operated in high-vacuum mode at an accelerating voltage of 20 kV.

#### 2.4.3 IR spectra of serum samples

The IR spectra of animal sera were analyzed using ATR-FTIR with the FT-IR/FT-NIR Spectrum 400 spectrometer from PerkinElmer (Waltham, MA, USA) as previously described (44). Fourier-transform infrared spectroscopy (FTIR) spectra were coupled with Attenuated Total Reflectance (ATR). To perform IR measurements, a Nicolet 6700 spectrometer equipped with a deuterated triglycine sulphate (DGTS) detector was employed. The spectra were obtained by adding 64 scans at a 2 cm^−1^ resolution. Serum samples were preserved at -80°C until analysis. Before measurement, the serum samples were thawed at 20°C using a CH-100 thermoblock (BIOSAN, Riga, Latvia) and shaken for 30 seconds with a LabDancer vario (IKA, Staufen im Breisgau, Germany). All measurements were conducted at 20°C with stable air humidity. The ATR crystal was cleaned with 95% alcohol before each measurement, then a baseline measurement was taken. One microliter of pure protein: (C - reactive protein (CRP), tumor necrosis factor - (TNF)-α, or immunoglobulins - (Ig)G), used as a standard or serum, was placed on the spectroscope crystal using a disposable, sterile pipette tip and allowed to dry as the water evaporated. The intensity of a band was used to assess the amount of evaporated water in the 3400–3200 cm^-1^ range. It was estimated that water had evaporated when the band intensity at 3300 cm-¹ dropped to approximately 40% of its initial value. Under these conditions, water evaporation took about 5 minutes. The high-water content in the sample reduces the intensity of the bands across the IR spectrum. After most of the water was removed, the spectrum of the residual material on the ATR crystal was recorded. IR spectra were captured in the wavenumber range of 4000–650 cm¹ with a resolution of 1 cm¹, then preprocessed in two steps: (a) calculating the first derivative using a five-point stencil, and (b) normalizing to the range of 0.1.

#### 2.4.4 qPCR

The *H. pylori* status in guinea pigs was assessed through quantitative PCR (qPCR). Postmortem gastric tissues from infected and control guinea pigs were homogenized, and total DNA was extracted using a commercial kit (Bead-Beat Micro AX Gravity, A&A Biotechnology, Gdańsk, Poland) following the manufacturer’s instructions. The isolated DNA was used as a template for qPCR reactions with SYBR Green dye detection. Primers were designed for the *H. pylori cagA* gene (encoding CagA protein) (F: 5’-ATAATGCTAAATTAGACAACTTGAGCGA-3’, 5’-TTAGAATAATCAACAAACATCACGCCAT-3’. Reference primers for guinea pig housekeeping genes ACTB and PPIA were included for normalization. qPCR mixtures were prepared with PowerUp™ SYBR™ Green Master Mix (Applied Biosystems™) and run on a thermocycler: initial denaturation at 95°C for 10 minutes, then 40 cycles at 95°C for 30 seconds, followed by annealing and extension at 58°C for 45 seconds. Melting curve analysis was performed post-amplification to verify PCR specificity. Relative quantification of gene copies was calculated using the ^ΔΔ^Ct method, comparing target gene Ct values to reference gene Ct values and expressing results relative to the control group.

### 2.5 Proceeding of gastric tissue

The stomach was incised along with the greater curvature, and its contents were removed. The tissue was washed three times with sterile phosphate-buffered saline (PBS) to remove any residual material. Subsequently, the gastric tissue (antrum part) and spleen were sectioned into small pieces and fixed in formalin for histological processing. The tissues were then embedded in paraffin and sectioned at 3 µm on a microtome.

#### 2.5.1 Immunohistochemical staining

Gastric tissue specimens were deparaffinized and rehydrated by immersion in increasing concentrations of alcohol, followed by rinsing in distilled water. The antigens were unmasked by heating tissue fragments for 20 min in a Citrate-Based solution at pH 6.0. Specimens were blocked with 5% bovine serum albumin (BSA) (Merck KGaA, Darmstadt, Germany) in PBS for 1h at room temperature. The tissues were then stained with primary rabbit monoclonal antibodies anti-sIgA (400x diluted in 1% BSA) (Invitrogen, Thermo Fisher Scientific, Waltham, MA, USA) or mouse anti-F40/80 antibodies (for detection tissue macrophages) (100x diluted in 1% BSA) (Invitrogen, Thermo Fisher Scientific, Waltham, MA, USA) (4°C, 18 hours), then tissues were washed three times with PBS, and secondary antibodies fluorescently labeled with AlexaFluor 488 or AlexaFluor 647, 2000x diluted (Invitrogen, Thermo Fisher Scientific, Waltham, MA, USA) were applied for 2h, room temperature. Excess antibodies were washed three times with PBS, and the cell nuclei were counterstained with 4’, 6-diamidino-2-phenylindole (DAPI) (1 μg/mL, 10 minutes, room temperature). Excess dye was washed off as above, and the tissue was mounted in mounting medium (Thermo Fisher Scientific, Waltham, MA, USA). The tissues were then imaged in a confocal microscope (MICA WideFocal Live Cell) using a 10x/0.32, 40x W/1.1 water immersion objective at the appropriate wavelengths for DAPI (358 nm excitation, 461 nm emission), AlexaFluor488 (490 nm excitation, 525 nm emission), or AlexaFluor 647 (650nm excitation, 671 nm emission). The Leica Application Suite X (LAS X) software (Leica Microsystems, Frankfurt, Germany) was used for cell imaging. Three independent experiments were performed in triplicate for each experimental variant. sIgA was quantified by fluorescence intensity. Analyzed tissue area 1 cm x 1 cm; 5 fields from each tissue.

### 2.6 Splenocyte proliferation

The spleen (red and white pulp; a piece of 2 cm x 2 cm) was cut into small pieces, homogenized using a glass homogenizer, and centrifuged (300×g, 10 min). According to the manufacturer, erythrocytes were lysed using the commercial RBC Lysis Buffer (Invitrogen, Thermo Fisher Scientific, Waltham, MA, USA). Spleen leucocytes 5×10^6^ cell/mL (100 µL/well) were added into the black well of a 96-well plate and were cultured in total 72h, 37°C, 5%CO_2_ in the cell growing medium (control) or in the presence of following components for 24h: *H. pylori* CCUG 17874 antigenic complex called a glycine acid extract (GE) (10 µg/mL), which was extracted using 0.2 M glycine buffer, pH 2.2, as previously described (47), LPS *H. pylori* CCUG 17874 (obtained courtesy of AP. Moran), or *E. coli* LPS derived from the O55:B5 strain (Merck KGaA, Darmstadt, Germany), (25 ng/mL), or *M. bovis* BCG (SYNTHAVERSE, Lublin, Poland), Brazilian Moreau substrain, in the multiplicity of infection (MOI): 10:1, or phytohemagglutinin (PHA), 2 µg/mL (Merck KGaA, Darmstadt, Germany), as a positive control. Stimulants were used in a total volume of 100 µL. Concentrations of individual stimulants were previously adjusted (15, 16, 27, 41, 48–51).

Proliferation of spleen lymphocytes was determined after 24h of cell stimulation using a commercial CyQUANT™ Cell Proliferation Assay (Invitrogen, Thermo Fisher Scientific, Waltham, MA, USA). The cells were then washed with PBS and frozen at −80 °C. Before testing, samples were thawed at room temperature and then lysed in a buffer containing CyQUANT-Red dye, prepared according to the manufacturer’s instructions. Fluorescence was measured at an emission wavelength of 782 nm and an excitation wavelength of 805 nm using a SpectraMax i3x Multi-Mode Microplate Reader (Molecular Devices, San Jose, CA, USA). Results were shown as a relative fluorescence units (RFU) ratio of studied samples vs. control. Three independent experiments were performed in triplicate for each experimental variant.

#### 2.6.1 Assessment of germinal centers

Spleens, which were isolated from the animals under the study, were routinely stained with H&E, and lymphoid follicles containing germinal centers (GC) were assessed. The analyzed field size was 5 cm x 5 cm with 3 fields of view per tissue.

### 2.7 Isolation of bone marrow-derived macrophages

The bone marrow was isolated from the tibias and femurs of *Cavia porcellus* as previously described (31, 48). Briefly, the bone marrow was centrifuged at 300×g for 10 minutes, washed three times with PBS, and cultured in cRPMI medium at 37°C with 5% CO_2_ for 1h. The non-adherent cells were then washed away, and immature macrophages were allowed to undergo maturation for 72h under the same conditions. Next, macrophages were detached, followed by two washes. The macrophages were adjusted to a density of 5×10^5^ cells/mL in cRPMI for subsequent experiments. BMDM were used to assess phagocytosis of fluorescently labelled *E. coli particles* (Vibrant Phagocytosis Assay Kit, Thermo Fisher Scientific, Waltham, MA, USA). The concentrations of TNF-α, IL-8, and IL-10 were determined in cell supernatants from 24 h cultures by ELISA. Moreover, Histone H3 Lysine 4 (H3K4) methyltransferase methylation was evaluated in a colorimetric assay. The surface deposition of CD11b integrin was assessed by immunofluorescence.

#### 2.7.1 BMDM CD11b staining

To assess surface CD11d on BMDM, cells were treated with a monoclonal mouse anti-CD11d antibody at a 1:200 dilution in 1% BSA/PBS (18h, 4°C). The BMDM were then treated with the secondary antibodies: goat anti-mouse immunoglobulins labelled with Alexa Fluor 488 (Invitrogen, CA, USA), diluted 1:200 in PBS containing 1% BSA – 1% BSA/PBS (2h, room temp.). The intensity of fluorescence was measured using a multifunctional reader, SpectraMax i3 (Molecular Devices, San Jose, CA, USA), at the appropriate wavelengths: Alexa Fluor 488 (excitation 495 nm, emission 519 nm). Three independent experiments were performed in triplicate for each experimental variant.

#### 2.7.2 Cytokine ELISA

BMDM isolated from the guinea pigs under the study were cultured as described above. Then the cell culture supernatants were collected for the estimation of selected cytokines by the ELISA (Invitrogen, Thermo Fisher Scientific, Waltham, MA, USA): IL-8 and TNF-α (sensitivity 1 pg/mL), and IL-10 (sensitivity 5 pg/mL), according to the manufacturer’s procedure.

#### 2.7.3 Phagocytosis and H3K4 methylation

Phagocytosis of fluorescently labelled *E. coli* (K-12 strain) particles by BMDM was assessed using the Vibrant Phagocytosis Assay Kit (ThermoFisher Scientific, Waltham, USA), as recommended by the manufacturer. Suspension of guinea pig BMDM (5×10^6^ cells/mL) was applied to the wells of a 96-well cell culture plate (100 μL/well). The fluorescence of engulfed particles was measured after 1h using a multifunctional reader, SpectraMax i3 (Molecular Devices, San Jose, CA, USA), with excitation at 495 nm and emission at 515 nm. The intensity of phagocytosis was shown as RFU (Relative Fluorescence Units). Three independent experiments were performed in triplicate for each experimental variant.

Nuclear BMDM extracts were prepared and used for colorimetric assessment of histone H3 lysine 4 methyltransferase methylation according to the manufacturer’s procedure (Abcam, Cambridge, UK). Absorbance was measured using a multifunctional reader, SpectraMax i3 (Molecular Devices, San Jose, CA, USA), at 450 nm.

#### 2.7.4 CD34, CD117 and Ly6G staining

Isolated BMDM, at a density of 3×10^5^ cells in 300 µL 1% BSA/PBS, were added to glass slides for obtaining cytospin-like preparations using a cytological centrifuge (MPW, Med. Instruments in Warszawa, Poland). The cells were fixed in 4% paraformaldehyde (PFA) for 30 minutes, followed by a 5-minute washing in PBS, then transferred to cold 100% methanol and subsequently blocked with 5% BSA/PBS for 60 min. Next, the slides were washed in PBS and subjected to immunofluorescent staining of the cluster of differentiation (CD) surface molecules CD34 and CD117, or Ly6G, using specific monoclonal antibodies fluorescently labelled (diluted 100x in 1% BSA/PBS) (Thermo Fisher Scientific, Waltham, MA, USA). The slides were kept in a humid chamber (4°C, 18h), and the cells were washed three times with PBS and then stained with the fluorescently labelled (AlexaFluor 488 or AlexaFluor 647, 2000x diluted) secondary antibodies (Invitrogen, Thermo Fisher Scientific, Waltham, MA, USA), for 2h at room temperature. Excess antibodies were washed out three times with PBS, and the cell nuclei were counterstained with 4’, 6-diamidino-2-phenylindole (DAPI) (1 μg/mL, 10 minutes, room temperature). Excess dye was washed out as above, and the cells were mounted in mounting medium (Thermo Fisher Scientific, Waltham, MA, USA). The BMDM were visualized in a fluorescence microscope (Zeiss, Axio Scope, A1, Oberkochen, Germany) using 20x/0.32, objective at the appropriate wavelengths for DAPI (358 nm excitation, 461 nm emission), AlexaFluor488 (490 nm excitation, 525 nm emission), and AlexaFluor 647 (650nm excitation, 671 nm emission). Results were presented as a percentage (%) of CD34, CD117, or Ly6G-positive cells vs. all nucleated cells. Three independent experiments were performed in triplicate for each experimental variant.

### 2.8 Statistical analysis

All graphs and statistical analyses were conducted using GraphPad Prism 10.2.2 for Windows (GraphPad Software, California, USA). The Shapiro-Wilk and D’Agostino-Pearson tests were used to assess the normality of both the data and the residuals, and to examine the Q-Q plots. The Brown-Forsythe test was used to determine equality of variances across groups. Data presentation reflects median ± range, with statistical significance defined as p < 0.05.

## 3 Results

Results were obtained for animal groups receiving only *H. pylori*, only CHI MPs modified with GclNAc (G) or Pluronic F127 (P) loaded with *M. bovis* BCG, or both types CHI MPs, or both types CHI MPs and then inoculated with *H. pylori*. Animals receiving *Brucella* broth served as controls. Results for G/P variants of CHI MPs loaded with *M. bovis* BCG administered to animals simultaneously are included in the main manuscript, while results for animals treated with the G or P variants of CHI MPs separately are included in the supplementary.

### 3.1 *H. pylori* status

**Figure 1:**
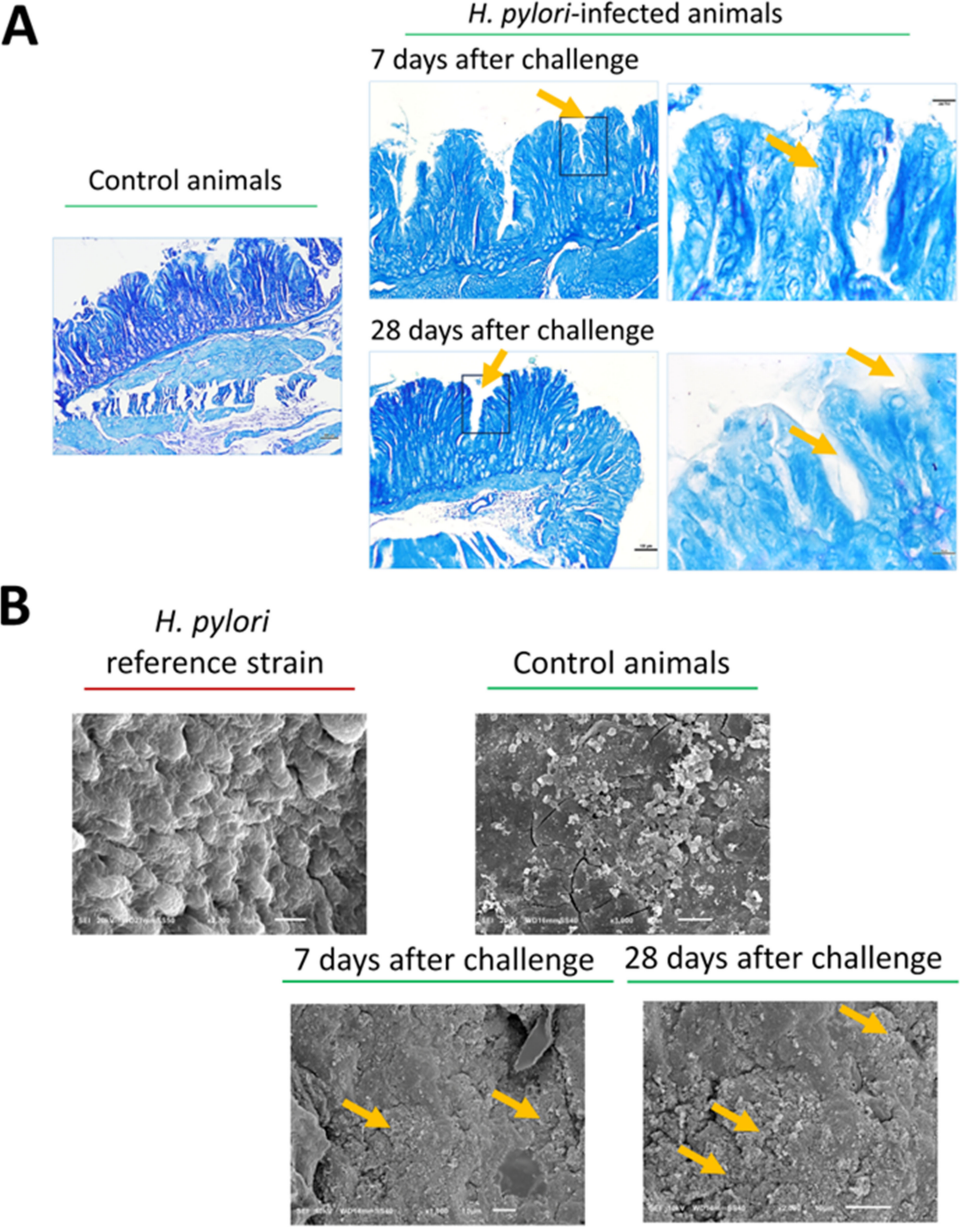
Gastric tissue histological and SEM images. **(A)** Representative microscopic images of Giemsa-stained gastric tissue from control and *H. pylori*-inoculated guinea pigs, obtained using a Nikon ECLIPSE 50i light microscope with a Nikon Plan 10x/0.25 Ph1 DR WD 10.5 or Nikon Plan 100XA/1.25 Oil WD 0.2 objective. The marked box and arrows indicate the localization of *Helicobacter-*like organisms. **(B)** - Representative scanning electron microscope (SEM) images of gastric tissue from control and *H. pylori*-inoculated guinea pigs. Yellow arrows indicate the localization of *H. pylori* in the examined tissue.

**Figure 2:**
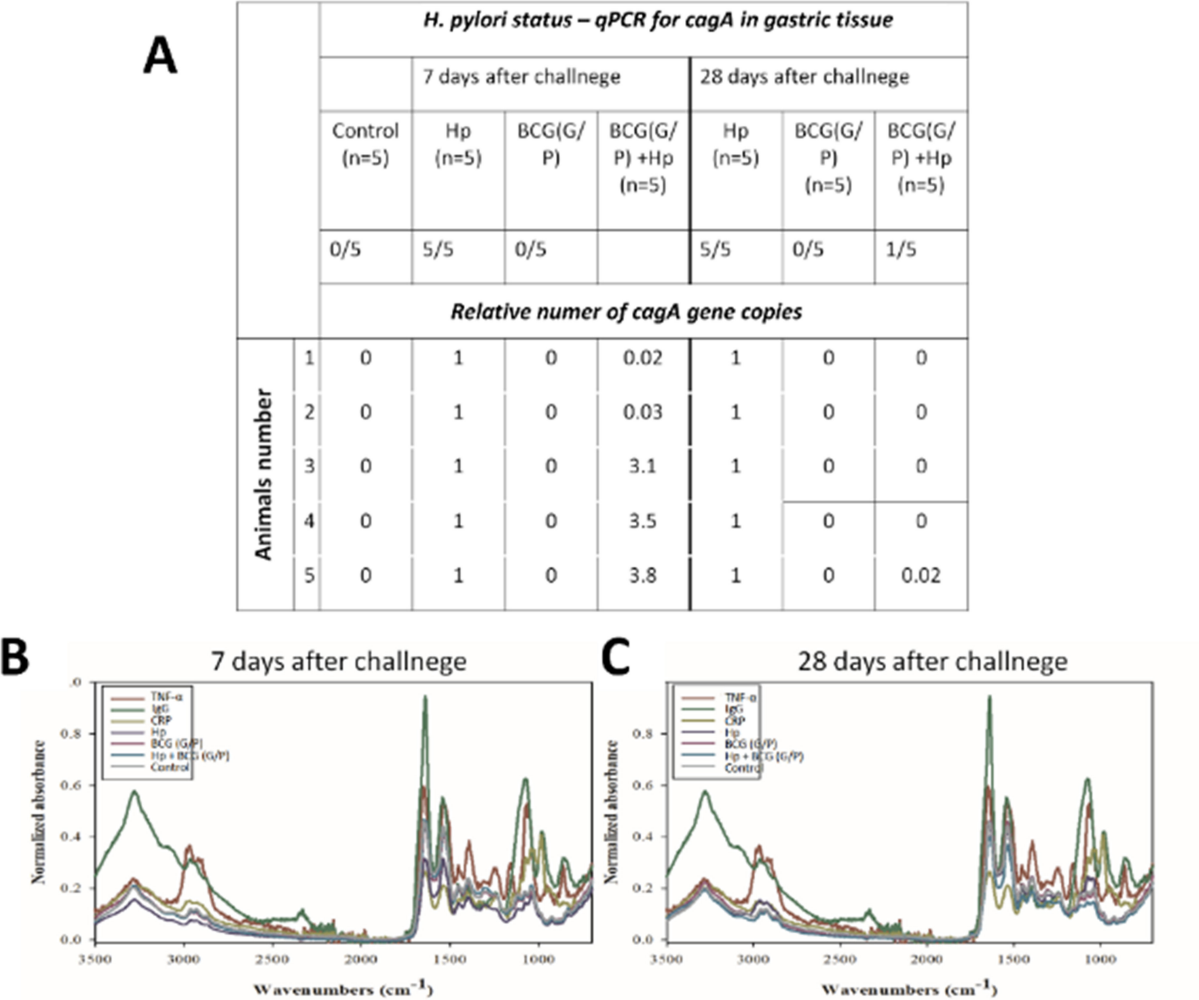
Animal *H. pylori* status. Quantitative polymerase chain reaction (qPCR) for *H. pylori cagA* gene **(A)**; Fourier transform infrared spectroscopy (FTIR) spectra of animal serum samples **(B, C)**. Hp, *H. pylori*; BCG, *M. bovis* BCG; G/P MPs, chitosan microparticles (CHI MPs) modified with N-acetylglucosamine (G, GlcNAc) or Pluronic F127 (P) and loaded with *M. bovis* BCG. Tumor Necrosis Factor (TNF-α); C-reactive protein (CRP). Animals received: only *H. pylori*, only chitosan microparticles (CHI MPs) loaded with *M. bovis* BCG, simultaneously both CHI MPs variants of CHI MPs - BCG (G/P) or such CHI MPs and then were inoculated with *H. pylori.* TNF-α, tumor necrosis factor alpha; IgG, class G immunoglobulin; CRP, C-reactive protein; Hp, *H. pylori*.

In animals inoculated with *H. pylori*, infection status was confirmed by histological examination, SEM analysis (**Figures 1A, B**), and qPCR for the detection of *cagA* gene in the gastric tissue (**Figure 2A**). Moreover, infrared spectra of serum samples were analyzed (**Figures 2B, C**). As shown in **Figures 1A**, in the gastric tissue of guinea pigs inoculated only with *H. pylori*, 7 days (5/5) or 28 days (5/5) from the last *H. pylori* dose, Helicobacter-like organisms (HLO) were detected in specimens stained with Giemsa stain solution and by SEM imaging **Figures 1A, B**. In animals inoculated with *H. pylori* alone, or in those receiving the studied CHI MPs loaded with *M. bovis* BCG and then inoculated with *H. pylori*, the infection was quantified by qPCR (**Figure 2A**). In animals treated with G/P CHI MPs loaded with *M. bovis* BCG, the colonization of gastric tissue by *H. pylori* was significantly decreased vs. animals inoculated only with *H. pylori*, as shown by the relative number of *cagA* gene copy (**Figure 2A**).

FTIR spectra of biological samples can be categorized into several component groups based on their characteristic absorption bands within specific wavenumber ranges: W1—fatty acids (3000 cm^-1^ to 2800 cm^-1^), W2—peptides and proteins (1800 cm^-1^ to 1500 cm^-1^), W3—proteins, phosphate-carrying compounds, and fatty acids (1500 cm^-1^ to 1200 cm^-1^), W4—carbohydrates (1200 cm^-1^ to 900 cm^-1^). Window W5 (wavenumber range of 900 cm^-1^ to 750 cm^-1^) corresponds to specific peaks unique to the biological samples (48). Based on our previous study (52) we detected unique wavenumbers (1552 cm^-1^, 1541 cm^-1^ and 1630 cm^-1^ – peptides and protein region, 1394 cm^-1^, 1395cm^-1^,1400cm^-1^, 1412cm^-1^, 1420cm^-1^ - proteins, phosphate-carrying compounds and fatty acid region, 1061cm^-1^, 1105 cm^-1^- carbohydrates region) in the IR spectrum of serum samples of studied guinea pigs correlating with *H. pylori* infection, 7 or 28 days after the last inoculation of animals with *H. pylori* (**Figures 2B, C**).

Additionally, in the FTIR analysis, we used the following standards: CRP and TNF-α proteins, as well as guinea pig IgG immunoglobulins. In the FTIR spectra we identified the following wave numbers corresponding to: IgG 571 cm^-1^, 1020 cm^-1^, 1120 cm^-1^, 1580 cm^-1^, 1690 cm^-1^, 3320 cm^-1^, reflecting an increased antibody levels as well as TNF-α 574 cm^-1^, 1110 cm^-1^, 1700 cm^-1^, 2990 cm^-1^ and CRP 547 cm^-1^, 1030 cm^-1^, 1080 cm^-1^, 1580 cm^-1^, 1680 cm^-1^, which both are inflammatory markers (**Figures 2B, C**). In *H. pylori*-infected animals, increased maxima for the absorbance bands corresponding to CRP, TNF-α and IgG are shown, while after administration of G/P CHI MPs loaded with *M. bovis* BCG to guinea pigs, the peaks corresponding to the wave numbers of CPR, TNF-α, and IgG were decreased 28 days after the last *H. pylori* dose, and were close to the maxima for control animals.

### 3.2 Gastric tissue macrophage infiltration

**Figure 3:**
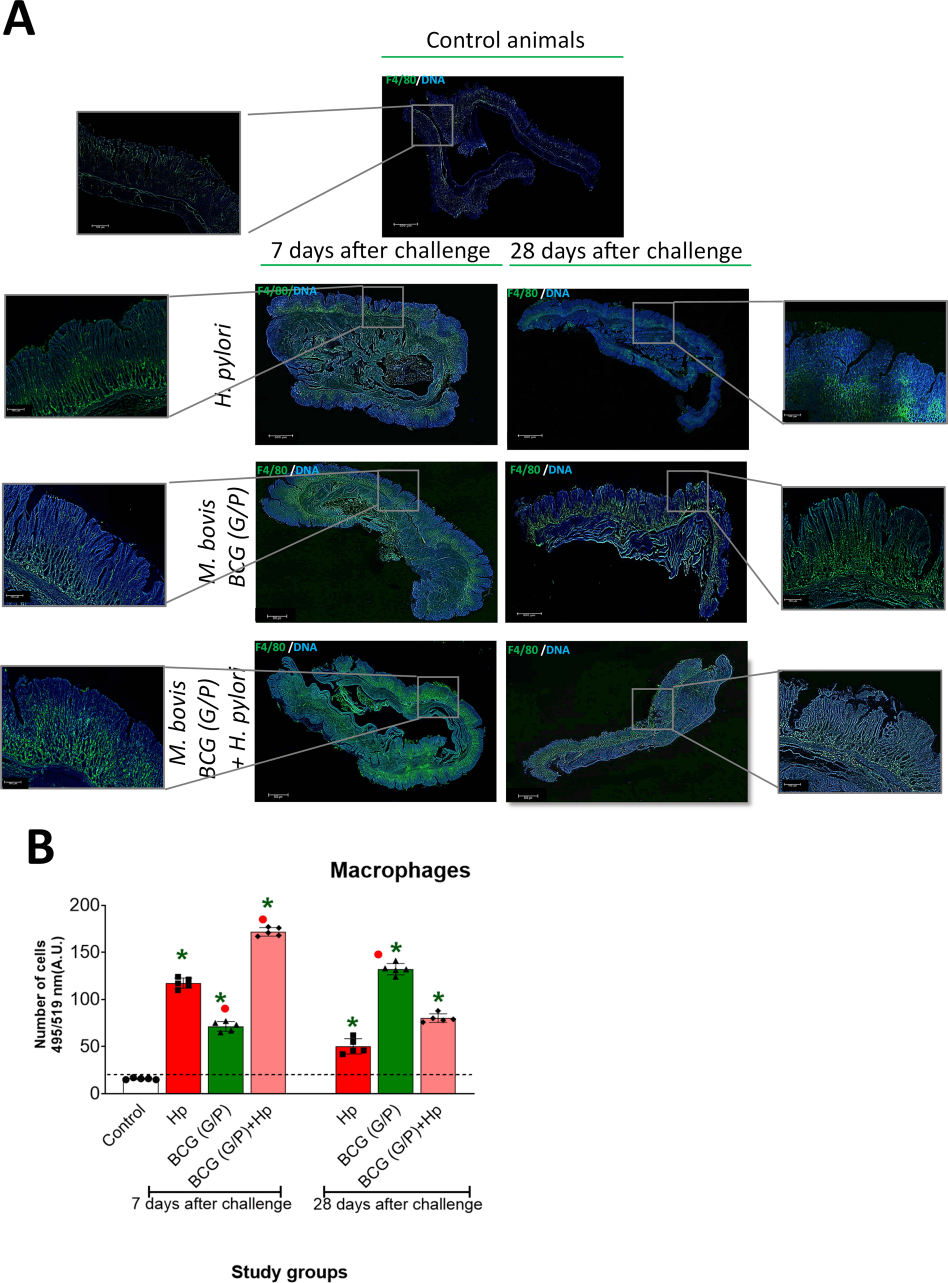
Gastric tissue macrophage infiltration. **(A)** Representative images of macrophage infiltration (green) in gastric tissue specimens of guinea pigs under this study. Macrophages were stained with primary mouse anti-F4/80 antibodies, followed by staining with Alexa Fluor 488-labelled anti-mouse immunoglobulin antibodies. Cell nuclei were stained with 4’, 6-diamidino-2-phenylindole dihydrochloride (DAPI). The images were observed under a confocal microscope at appropriate wavelengths: for DAPI, 345 nm (excitation) and 455 nm (emission); for Alexa Fluor 488, 490 nm (excitation) and 525 nm (emission), magnification 10x or 40x, water immersion (Microhub Leica MICA). **(B)** - The number of macrophages was assessed by assessing the tissue area 1 cm x 1 cm; 5 fields from each tissue. Macrophage assessment scale: grade 1 - up to 15 macrophages; grade 2 – up to 50 macrophages, mainly below the muscularis mucosa; grade 3 – up to 75 macrophages, below and above the muscularis mucosa; grade 4 – above 100 macrophages through the whole tissue. Results are presented as the number of macrophages (*arbitrary units* A.U.) ± range of three independent experiments. Statistical significance for p <0.05 in the non-parametric Mann-Whitney or Kruskal-Wallis U test. * Animals non-treated (control groups) vs. animals treated with chitosan microparticles (CHI MPs) loaded with *M. bovis* BCG or inoculated with *H. pylori* or first receiving such CHI MPs and then inoculated with *H. pylori*. • Animals treated with *H. pylori* vs. animals treated with CHI MPs loaded with *M. bovis* BCG or first receiving CHI MPs loaded with *M. bovis* BCG and then inoculated with *H. pylori* (comparison by treatment times). Animals revised: only *H. pylori*, only chitosan microparticles (CHI MPs), variant G and P simultaneously, loaded with *Mycobacterium bovis* BCG or CHI MPs and then were inoculated with *H. pylori*. *M. bovis* BCG (G/P) - chitosan microparticles (CHI MPs) modified with GlcNAc - N-acetylglucosamine (G) or with Pluronic F127 (P) loaded with *M. bovis* BCG.

Tissue macrophages in animals studied were stained by immunofluorescence using specific anti-F4/80 primary antibodies and fluorescently labelled secondary antibodies, and identified by confocal microscopy. The following gradation of macrophage infiltration was used: grade 1 – up to 15 macrophages in the imaged area; grade 2 – up to 50 macrophages mainly below the muscularis mucosa; grade 3 – up to 75 macrophages below and above the muscularis mucosa; grade 4 –above 100 macrophages throughout the whole tissue. In the gastric tissue of control animals uninfected with *H. pylori*, we detected single macrophages in the gastric tissue (grade 1), while in animals inoculated with *H. pylori*, after 7 days from the last inoculation, the macrophage infiltration was at grade 4, while after 28 days, at grade 2 (**Figures 3A, B**). Administration of G/P CHI MPs loaded with *M. bovis* BCG alone induced macrophage infiltration at grade 2 after 7 days and at grade 4 after 28 days. In guinea pigs inoculated first with G/P CHI MPs loaded with *M. bovis* BCG and then with *H. pylori*, macrophage infiltration after 7 days post-infection was at grade 4, while after 28 days from *H. pylori* inoculation, macrophage infiltration reached grade 2 (**Figures 3A, B**). Infiltration of macrophages was significantly higher in animals receiving G/P CHI MPs loaded with *M. bovis* BCG and infected with *H. pylori* (above 100 cells), after 7 days from inoculation, compared to animals which were exposed to G or P variant of CHI MPs loaded with *M. bovis* BCG and then infected with *H. pylori*, after 7 days from the last dose (up to 75 cells) (**Supplementary Figures 1A, B**). In animals receiving simultaneously G/P CHI MPs with *M. bovis* BCG the macrophage infiltration after 28 days was higher (above 100 cells) as compared to macrophage infiltration in animals treated with P or G CHI MPs variants (50–75 cells) (**Supplementary Figures 1A, B**).

### 3.3 Bone marrow cell phenotyping (CD34, CD117 and Ly6G)

**Figure 4:**
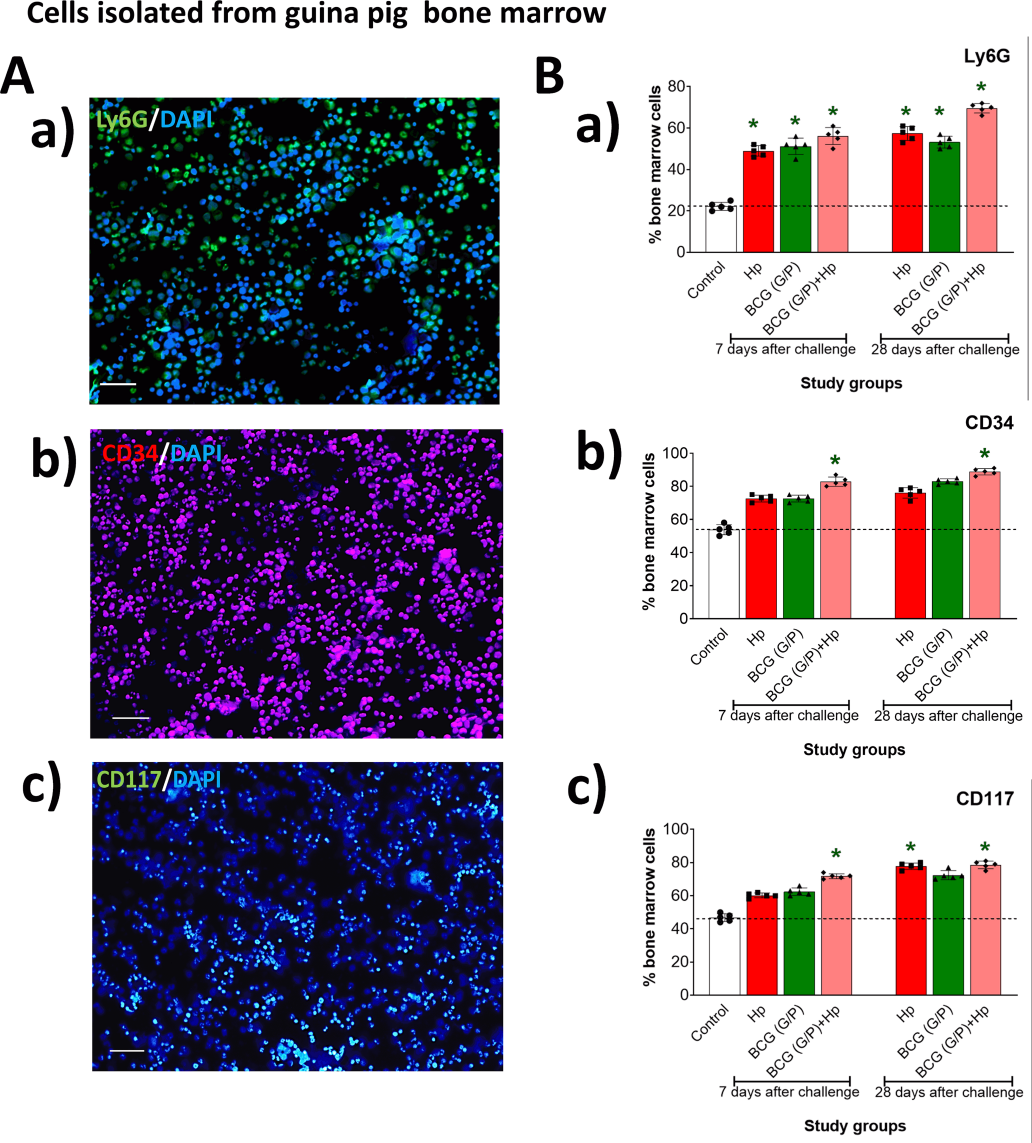
Phenotyping of bone marrow cells. **(A)** Representative images of Ly6G positive cells **(a)**, CD34 positive cells **(b)** and CD117 positive cells **(c)** in the bone marrow. Cells were stained with primary rabbit anti-Ly6G antibodies, anti-CD34, or anti-CD117 antibodies, and then with secondary antibodies fluorescently labelled Alexa Fluor 488 (green) or Alexa Fluor 568 (red). Cell nuclei were counterstained with 4’, 6-diamidino-2-phenylindole dihydrochloride (DAPI). Representative images were prepared under a fluorescence microscope (Zeiss, Axio Scope, A1, Oberkochen, Germany) using 20x/0.32 objective at the appropriate wavelengths for DAPI (358 nm excitation, 461 nm emission), AlexaFluor488 (490 nm excitation, 525 nm emission), AlexaFluor 647 (650nm excitation, 671nm emission). **(B)** Percentage (%) of CD34, CD117, or LY6G-positive cells vs. nucleated cells. Statistical significance for p <0.05 in the non-parametric Mann-Whitney or Kruskal-Wallis U test. * Animals non-treated (control groups) vs. animals treated simultaneously with G and P variant of chitosan microparticles (CHI MPs) loaded with *M. bovis* BCG, inoculated only with *H. pylori*, or first receiving CHI MPs loaded with *M. bovis* BCG and then inoculated with *H. pylori*. • animals inoculated with *H. pylori* vs. animals treated with MPs loaded with *M. bovis* BCG or first receiving CHI MPs loaded with *M. bovis* BCG and then inoculated with *H. pylori* (comparison concerning treatment time). Animals received only *H. pylori*, only chitosan microparticles (CHI MPs) loaded with *M. bovis* BCG, or such CHI MPs, and were then inoculated with *H. pylori.* BCG (G/P) – CHI MPs modified with N-acetylglucosamine (GlcNAc) or with Pulronic F127 (P), respectively and loaded with *M. bovis* BCG.

We analyzed the hematopoietic markers CD34, CD117 and Ly6G, representative for myelocytic precursors in the bone marrow of the studied guinea pigs, correlating with the proliferation and differentiation of monocytes, macrophages and neutrophils (**Figure 4**, **Supplementary Figure 2**). In animals inoculated with *H. pylori* alone or only with G/P variant of CHI MPs loaded with *M. bovis* BCG, or in animals receiving such CHI MPs and then inoculated with *H. pylori* the increased percentage of bone marrow cells stained positively for neutrophil Ly6G cell marker corelating with proliferation and maturation of these cell precursors was shown after 7 or 28 days from the last inoculation compared to control animals, with no difference between these two assessment points (**Figures 4Aa, Ba**). Concerning the CD34 cell marker correlating with the proliferation of monocyte/macrophage precursors, the increased percentage of cells stained positively with anti-CD34 antibodies was shown in the bone marrow of animals receiving G/P CHI MPs loaded with *M. bovis* BCG after 7 and 28 days from the last inoculation (**Figures 4Ab, 4Bb**). The increased percentage of bone marrow cells positively stained for CD117 tyrosine kinase, a receptor for Stem Cell Factor, was shown in animals inoculated with *H. pylori* alone after 28 days from the last inoculation (**Figures 4Ac, 4Bc**). An increased number of CD117 positively stained cells was also shown in the bone marrow of animals receiving G/P CHI MPs loaded with *M. bovis* BCG and then infected with *H. pylori* after 7 or 28 days from the last inoculation (**Figures 4Ac, 4Bc**). Similar tendencies were observed in animals exposed independently to G or P CHI MPs loaded with *M. bovis* BCG, or to such CHI MPs and then infected with *H. pylori* (**Supplementary Figure 2**).

### 3.4 BMDM cytokine profile

**Figure 5:**
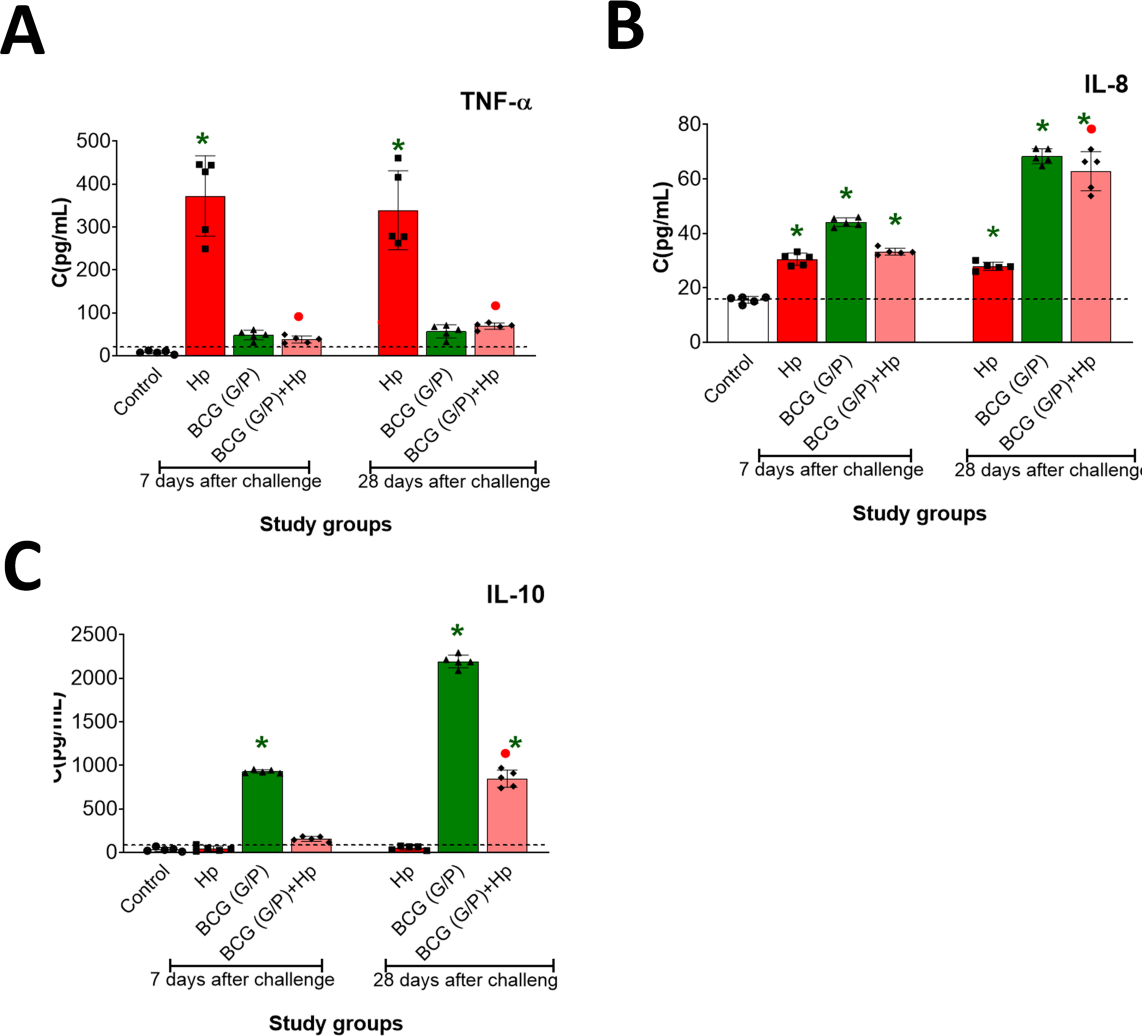
BMDM cytokine profile. The level of TNF-a **(A)**, IL-8 **(B)** and IL-10 **(C)** was estimated by the ELISA assay (sensitivity 1 pg/mL). Results are presented as mean ± range of three independent experiments. Statistical significance for *p* < 0.05 in the non-parametric Mann-Whitney or Kruskal-Wallis U test. * Animals non-treated (control group) vs. animals treated with chitosan microparticles (CHI MPs) loaded with *M. bovis* BCG, or with *H. pylori*, or first with CHI MPs and then infected with *H. pylori*. • animals treated with *H. pylori* vs. animals treated with CHI MPs loaded with *M. bovis* BCG or first receiving CHI MPs loaded with *M. bovis* BCG and then infected with *H. pylori* (comparison by treatment times). Animals revised: only *H. pylori*, only chitosan microparticles (CHI MPs) modified with N-acetylglucosamine (GlcNAc) (G) or with Pluronic F 127 (P) (CHI MPs variants were administered simultaneously) or first received such CHI MPs and then were infected with *H. pylori*.

In the guinea pigs under this study, we determined the secretion by BMDM proinflammatory cytokines, TNF-α and IL-8, as well as anti-inflammatory/regulatory IL-10. Only in animals, which were inoculated with *H. pylori* alone, after 7 or 28 days from the last inoculation the level of TNF-α was significantly increased in BMDM cultures (**Figure 5A**). In cultures of BMDM from animals inoculated only with *H. pylori* and those which were inoculated only with G/P CHI MPs loaded with *M. bovis* BCG as well as from guinea pigs which first received such CHI MPs and then were infected with *H. pylori* the level of IL-8 increased after 7 and 28 days compared to BMDM cultures from control animals (**Figure 5B**). The highest level of IL-8 was detected after 28 days in BMDM cultures from animals receiving G/P CHI MPs loaded with *M. bovis* BCG or those exposed first to such CHI MPs and then infected with *H. pylori* (**Figure 5B**).

Concerning the secretion of anti-inflammatory/regulatory IL-10, the level of this cytokine was significantly increased in cultures of BMDM from animals receiving only G/P CHI MPs loaded with *M. bovis* BCG (after 7 or 28 days of exposure) and in BMDM cultures from animals exposed to such CHI MPs and then infected with *H. pylori* (after 28 days post inoculation) (**Figure 5C**). Similar results were obtained for BMDM isolated from animals receiving G or P variant of CHI MPs loaded with *M. bovis* BCG, separately, or receiving CHI MPs and then *H. pylori* (**Supplementary Figures 3A–C**).

### 3.5 BMDM phagocytic activity and H3K4 methylation

**Figure 6:**
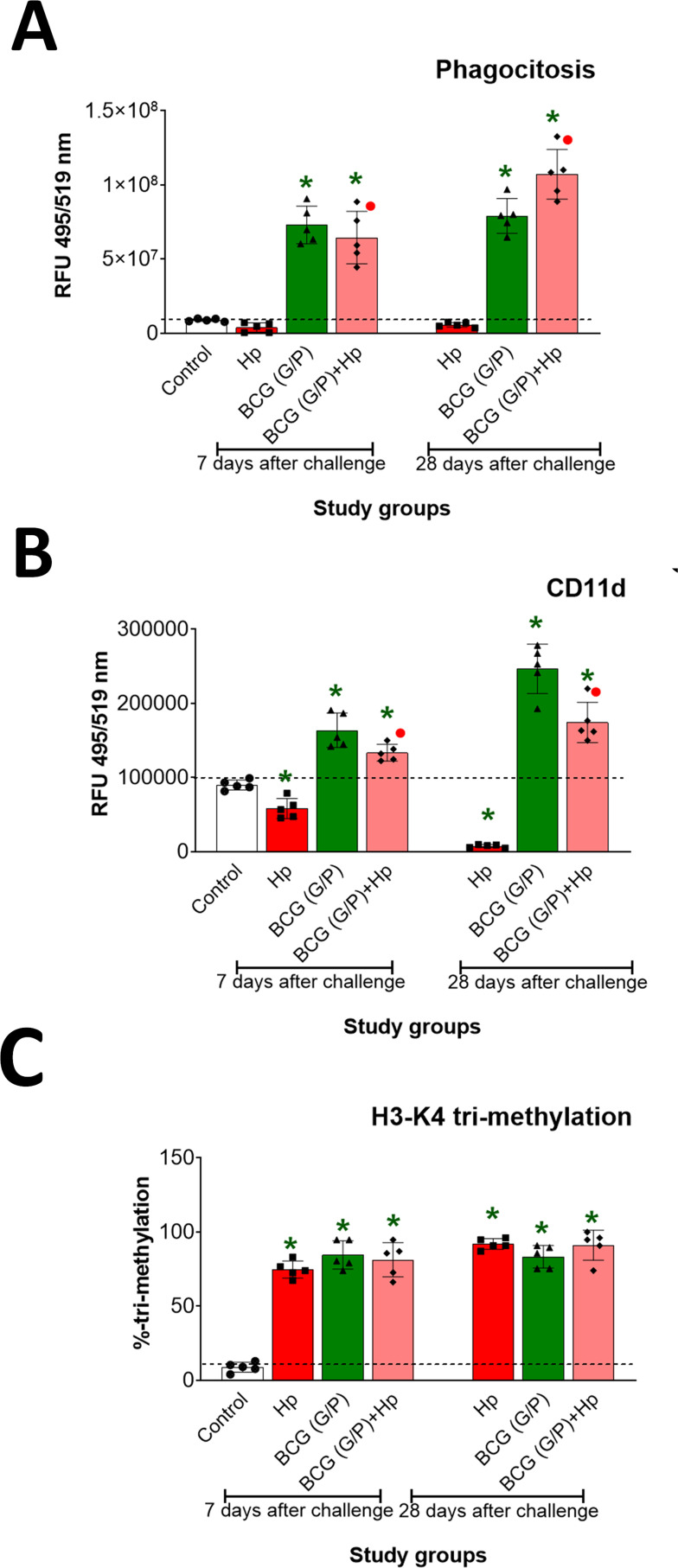
BMDM phagocytic activity and H3K4 methylation. **(A)** The phagocytic activity of guinea pig bone marrow-derived macrophages (BMDM) towards fluorescently labelled *E. coli* (Vybrant phagocytosis assay kit). **(B)** CD11d expression on BMDM. **(C)** - Methylation of histone H3 at lysine 4 (H3K4). Phagocytosis of *E. coli* fluorescently labelled particles by BMDM was measured using a multifunctional reader, SpectraMax i3 (Molecular Devices, San Jose, CA, USA), at an excitation wavelength of 495 nm and an emission wavelength of 515 nm. CD11d deposition was assessed by staining cells with primary mouse anti-CD11d antibodies, followed by secondary antibodies fluorescently labelled with AlexaFluor 488 (green) (490 nm excitation, 525 nm emission). Methylation of H3K4 was assessed using a colorimetric assay and expressed as the percentage of H3K4-positive cells. Results **(A–C)** are presented as ratios of median fluorescence units (RFU) ± range of three independent experiments. Statistical significance for p <0.05 in the non-parametric Mann-Whitney or Kruskal-Wallis U test. * Animals non-treated (control groups) vs. treated with chitosan microparticles (CHI MPs), simultaneously with G and P variant, loaded with *M. bovis* BCG, inoculated only with *H. pylori* or first receiving such CHI MPs and then inoculated with *H. pylori*. • Animals treated with *H. pylori* vs. animals treated with CHI MPs loaded with *M. bovis* BCG or first receiving CHI MPs loaded with *M. bovis* BCG and then inoculated with *H. pylori* (comparison by treatment times). Animals received: only *H. pylori*, only CHI MPs loaded with *M. bovis* BCG modified with N-acetylglucosamine (GlcNAc) (G) and CHI MPs loaded with *M. bovis* BCG modified with Pulonic F127 (P).

In this study, the phagocytic activity of BMDM isolated from the animals under the study was assessed *in vitro* using fluorescently labelled *E. coli* particles. Phagocytic activity of BMDM from animals inoculated only with *H. pylori* was at the level of control (BMDM from non-infected guinea pigs), 7 or 28 days from inoculation. In contrast, the ability to engulf *E. coli* particles by BMDM from animals receiving G/P CHI MPs loaded with *M. bovis* BCG alone was significantly increased in both assessment points (**Figure 6A**). Similarly, the phagocytosis of *E. coli* by BMDM from animals receiving such CHI MPs and then inoculated with *H. pylori* was significantly increased. The highest engulfment of *E. coli* particles was shown after 28 days from the last inoculation (**Figure 6A**). The increased engulfment of *E. coli* by BMDM from animals receiving only G/P CHI MPs loaded with *M. bovis* BCG or those additionally inoculated with *H. pylori*, 7 or 28 days from inoculation, was related to increased expression of CD11d integrin (**Figure 6B**).

Considering the modulatory activity of *M. bovis* BCG towards innate immune cells, we assessed H3K4 methylation in BMDM from the animals under the study. In all investigated groups: animals inoculated only with *H. pylori* or with G/P CHI MPs loaded with *M. bovis* BCG, or those which first received such CHI MPs and then were inoculated with *H. pylori*, the histone H3K4 methylation significantly increased as compared to BMDM from control animals; however, there was no difference between groups (**Figure 6C**).

Comparing the results for G or P variant of CHI MPs loaded with *M. bovis* BCG administered separately, we observed tendencies similar to simultaneous administration of G/P CHI MPs loaded with *M. bovis* BCG regarding CD11d expression and H3K4 methylation (**Supplementary Figures 4B, C**). However, BMDM from animals receiving G CHI MPs loaded with *M. bovis* BCG and then infected with *H. pylori* showed significantly increased phagocytic activity towards *E. coli* particles, when isolated 28 days from inoculation while BMDM from animals treated with P variant of MPs loaded with *M. bovis* BCG and inoculated with *H. pylori* showed significantly higher engulfment of *E. coli* particles 7 and 28 days from inoculation (**Supplementary Figure 4A**).

### 3.6 CHI MPs driven acquired immunity

#### 3.6.1 Gastric tissue sIgA

In animals under the study, we assessed the level of sIgA in gastric tissue and the number of lymphoid follicles with activated germinal centers (GC) in the spleen. The proliferation of splenocytes has also been assessed *in vitro*.

**Figure 7:**
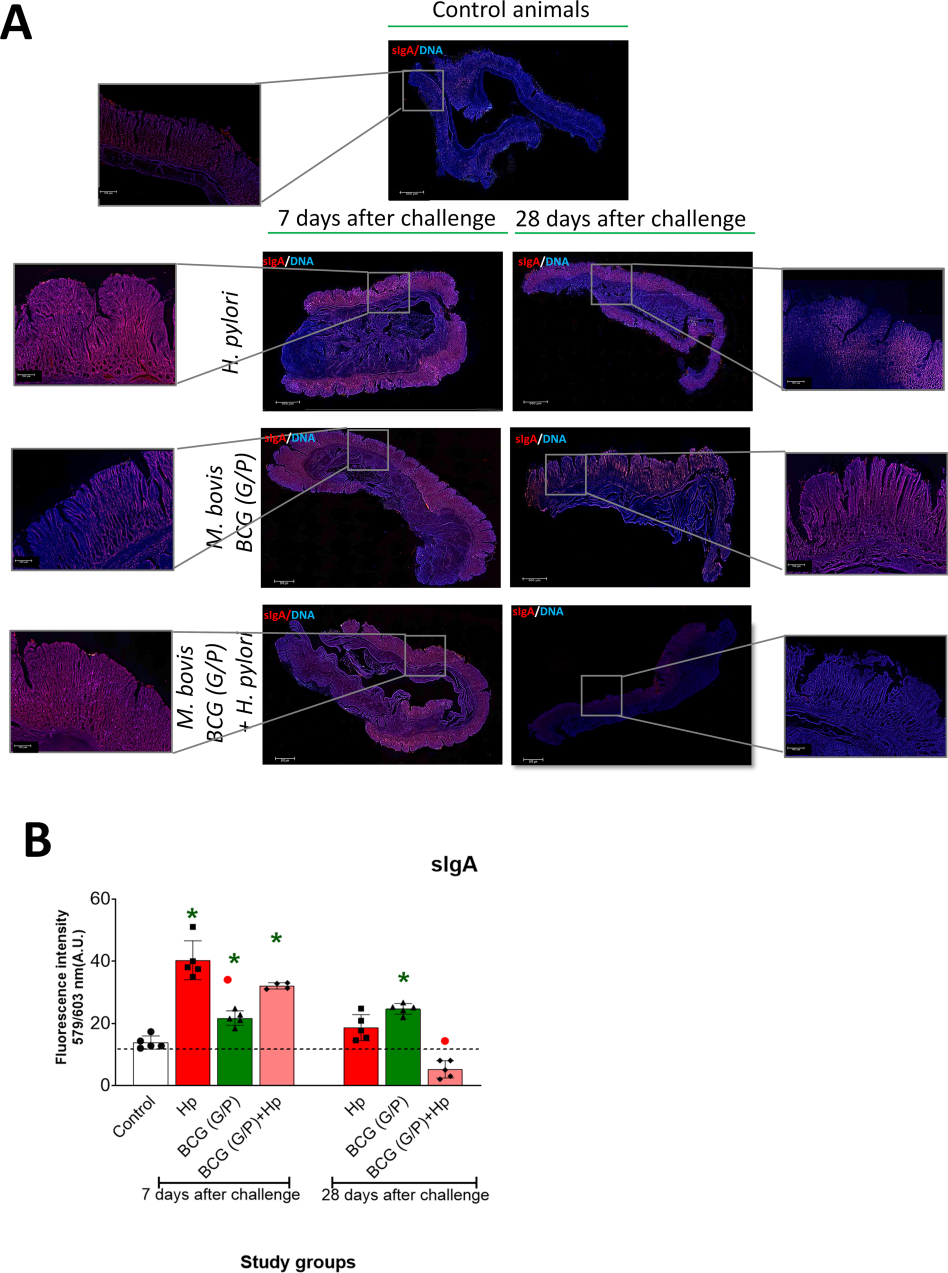
Gastric tissue sIgA. **(A)** Representative images of guinea pig gastric tissue specimens stained with anti-sIgA antibodies and then with secondary antibodies labelled AlexaFluor 647. Cell nuclei were counterstained with 4’, 6-diamidino-2-phenylindole dihydrochloride (DAPI). Representative images were prepared under a confocal microscope at appropriate wavelengths: DAPI: 345 (excitation) and 455 (emission); AlexaFluor 647: 650 nm (excitation) and 671 nm (emission), magnification 10x or 40x water immersion (Microhub Leica MICA). **(B)** - Level of sIgA fluorescence was assessed by screening of tissue area 1 cm x 1 cm; 5 fields from each tissue. sIgA assessment scale shown in arbitrary fluorescence units (AU): 1- up to 15 FU, 2- up to 25 FU, 3- up to 35 FU, 4-above 35 FU. A.U - arbitrary units; corresponding to relative fluorescence intensity. Results are presented as A.U. ± range of three independent experiments. Statistical significance for p <0.05 in the non-parametric Mann-Whitney or Kruskal-Wallis U test. * Animals non-treated (control groups) vs. treated with chitosan microparticles (CHI MPs), G and P variant simultaneously, loaded with *M. bovis* BCG or inoculated with *H. pylori* or first receiving such CHI MPs and then inoculated with *H. pylori*. • Animals treated with *H. pylori* vs. animals treated with CHI MPs loaded with *M. bovis* BCG or first receiving CHI MPs loaded with *M. bovis* BCG and then inoculated with *H. pylori* (comparison by treatment times). Animals revised: only *H. pylori*, only *M. bovis* BCG (G/P) - chitosan microparticles (CHI MPs) modified with N-acetylglucosamine (GlcNAc) (G) and chitosan microparticles (CHI MPs) modified with Pluronic F127 (P) loaded with *M. bovis* BCG or such MPs and then were infected with *H. pylori*.

We examined gastric tissue of studied animals for the presence of sIgA based on fluorescence intensity after staining the spleen specimens with specific anti-sIgA antibodies fluorescently labeled (**Figures 7A, B**). In animals exposed to *H. pylori* alone, the sIgA fluorescence in the gastric tissue was significantly enhanced after 7 days from the last inoculation (**Figures 7A, B**). In animals receiving only G/P CHI MPs loaded with *M. bovis* BCG the intensity of sIgA fluorescence after 7 or 28 days from animal exposure was not significantly increased compared to control animals. In guinea pigs, first exposed to G/P CHI MPs loaded with *M. bovis* BCG and then inoculated with *H. pylori*, fluorescence intensity measured 7 days after inoculation was significantly higher than in control animals. Its intensity was as high as in gastric specimens from animals receiving only *H. pylori*. By comparison, the intensity of sIgA fluorescence in gastric tissue from animals treated with G/P CHI MPs loaded with *M. bovis* BCG was at the level of control after 28 days from the last inoculation with *H. pylori* (**Figures 7A, B**). Comparing the effects induced independently by G or P variant of CHI MP loaded with *M. bovis* BCG the fluorescence of sIgA was significantly increased in animals receiving variant G CHI MPs after 7 or 28 days, while in animals receiving variant P CHI MPs the increased sIgA fluorescence was shown after 28 days from the last inoculation. There was only a tendency of increased fluorescence in the gastric tissue of animals exposed to G or P CHI MPs variant and then inoculated with *H. pylori* as compared to control, particularly after 28 days (**Supplementary Figures 5A, B**).

#### 3.6.2 Propagation of splenic germinal centers

**Figure 8:**
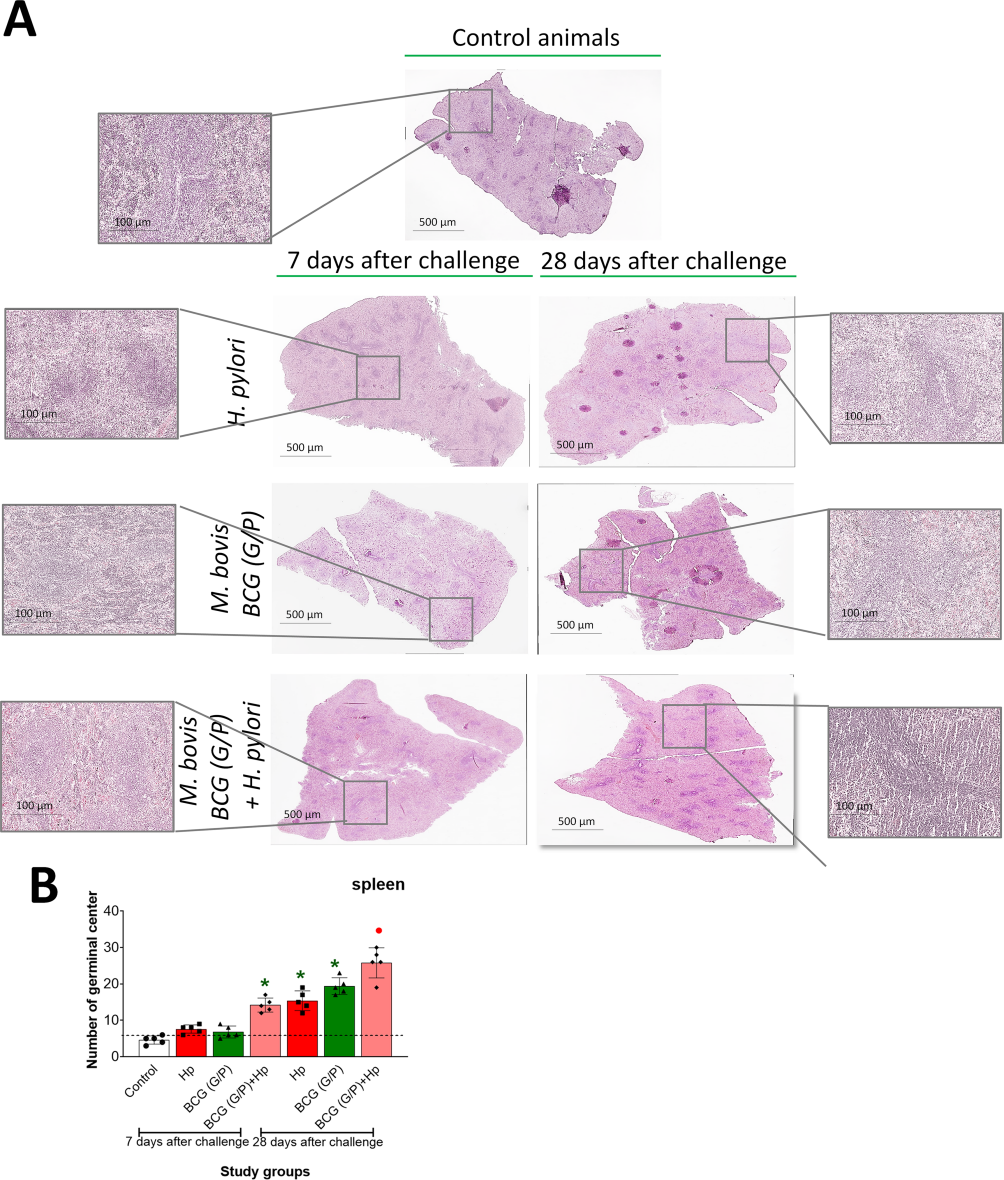
Propagation of splenic germinal centers. **(A)** - Representative images of spleen specimens of studied guinea pigs stained with H&E from a confocal microscope in transmitted light, magnification 10x, or 40x water immersion (Microhub Leica MICA). **(B)** - percentage of germinal centers in lymphoid follicles in the spleen sections. Results are presented as mean ± range of three independent experiments. In each section, 3 fields (5 cm x 5 cm) were assessed. Statistical significance for p <0.05 in the non-parametric Mann-Whitney or Kruskal-Wallis U test. * Animals non-treated (control groups) vs. treated with a mixture of chitosan microparticles (CHI MPs), modified with N-acetylglucosamine (GlcNAc) (G) or with Pluronic F127 (P) loaded with M. bovis BCG and then inoculated with H. pylori. • animals treated with *H. pylori* vs. animals treated with CHI MPs or *H. pylori*, or first receiving CHI MPs and then infected with *H. pylori* (comparison by treatment times). Animals revised: only *H. pylori*, only BCG (G/P) - chitosan microparticles (CHI MPs) variant G and P simultaneously or such CHI MPs and then were infected with *H. pylori*.

We used H&E-stained thin-layer sections of spleen tissue from the studied animals to assess the number of lymphoid follicles containing GCs, where B cells mature, replicate, and undergo mutations in genes encoding immunoglobulin variable domains. In animals inoculated with *H. pylori* alone or those receiving only the G/P variant of CHI MPs loaded with *M. bovis* BCG, after 7 days, the number of lymphoid follicles with GCs was at the level of control. (**Figures 8A, B**). Only in animals exposed to G/P CHI MPs and then inoculated with *H. pylori* the number of propagated GCs significantly increased. After 28 days of measurement, in animals inoculated with *H. pylori* alone or in those treated with G/P CHI MPs loaded with *M. bovis* BCG as well as in guinea pigs exposed to such CHI MPs and then inoculated with *H. pylori*, the number of lymphoid follicles with activated GCs was significantly increased (**Figures 8A, B**). The highest number of activated lymphoid follicles was in the last group of animals. Comparing the number of GCs in lymphoid follicles of animals receiving G CHI MPs or P CHI MPs loaded with *M. bovis* BCG we showed the significant GCs increase only in animals receiving variant G of CHI MPs, after 7 days (**Supplementary Figures 6A, B**). After 28 days, the increased number of activated lymphoid follicles was shown in the spleen of *H. pylori* infected animals, those receiving only G CHI MPs or P CHI MPs loaded with *M. bovis* BCG or in animals treated with such CHI MPs and then infected with *H. pylori* (**Supplementary Figures 6A, B**).

#### 3.6.3 Proliferation of splenocytes

To investigate whether inoculation of animals with *H. pylori* alone or pretreating them with CHI MPs loaded with *M. bovis* BCG or exposure to such CHI MPs and then inoculation with *H. pylori* can stimulate the lymphocyte expansion, we assessed proliferative activity of splenocytes from the studied animals in cell cultures *in vitro* in the presence of GE, an antigenic complex of *H. pylori* surface components, *H. pylori* LPS or *E. coli* LPS, or *M. bovis* BCG. Phytohemagglutinin was used as a positive control for assessing splenocyte proliferation.

**Figure 9:**
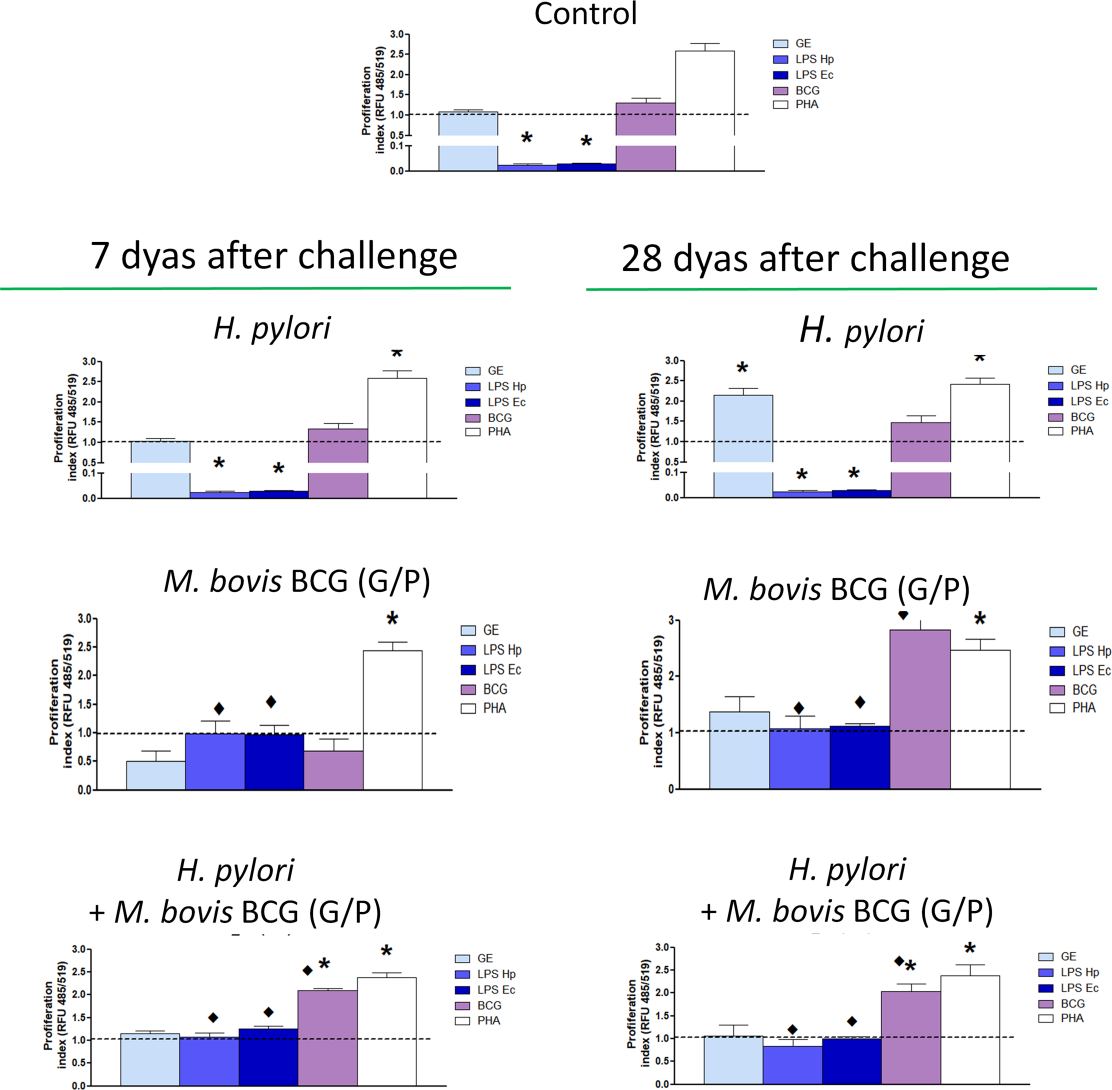
Splenocyte proliferation. For the proliferation assay the following splenocytes from *in vitro* cell cultures were used: untreated or treated for 24 h with *M. bovis* BCG (MOI 1:10), *H. pylori* glycine extracts - GE (10 μg/mL), *H. pylori* lipopolysaccharide - LPS (25 ng/mL), *E. coli* LPS (25 ng/mL), or phytohemagglutinin - PHA (2 µg/mL). Proliferation was determined using the Invitrogen™ CyQUANT™ Cell Proliferation Assay, and results were expressed as the number of cells in culture. Results are presented as mean ± range of three independent experiments. Statistical significance for p <0.05 in the non-parametric Mann-Whitney or Kruskal-Wallis U test. * Animals non-treated (control group) vs. animals treated with chitosan microparticles (CHI MPs), variant G and variant P, loaded with *M. bovis* BCG or inoculated only with *H. pylori*, or first receiving CHI MPs loaded with *M. bovis* BCG and then infected with *H. pylori*. • animals treated with *H. pylori* vs. treated with CHI MPs or *H. pylori*, or first receiving CHI MPs then infected with *H. pylori* (comparison by treatment times). Animals received: only *H. pylori*, only chitosan microparticles (CHI MPs) loaded with *Mycobacterium bovis* modified with N-acetylglucosamine (GlcNAc) (G), and CHI MPs loaded with *M. bovis-*BCG modified with Pluronic F127 (P), or first were infected with *H. pylori*, and then were infected with *H. pylori*.

Spleen lymphocytes from all groups of animals proliferated in the presence of PHA (**Figure 9**). In animals inoculated with *H. pylori*, only splenocytes isolated after 28 days, but not after 7 days, responded to *H. pylori* GE by proliferation. Proliferating activity of splenocytes, from animals after 7 or 28 days from inoculation with *H. pylori* and exposed *in vitro* to *H. pylori* LPS or *E. coli* LPS, was below the proliferating activity of cells in culture medium alone (**Figure 9**). Splenocytes from animals which were inoculated only with *H. pylori* did not proliferate *in vitro* in the presence of *M. bovis* BCG (**Figure 9**). Concerning animals inoculated only with G/P CHI MPs loaded with *M. bovis* BCG, only splenocytes isolated 28 days from inoculation responded *in vitro* by increased proliferation in the presence of *M. bovis* BCG. Interestingly, the proliferative response of this group of splenocytes to bacterial LPS was greater than response of cells obtained from animals inoculated with *H. pylori* alone (**Figure 9**). Similarly, proliferative activity of splenocytes from animals exposed to G/P CHI MPs loaded with *M. bovis* BCG and then infected with *H. pylori* was upregulated 7 or 28 days post-inoculation in response to mycobacteria added to cell culture. It was restored in response to bacterial LPS (**Figure 9**). Similar proliferation trends showed splenocytes of animals receiving G or P CHI MPs variant loaded with *M. bovis* BCG (**Supplementary Figure 7**).

## 4 Discussion

In this study, we used the guinea pig model (42) for *per os* inoculation with *M. bovis* BCG delivered by CHI MPs as a potential immunomodulator facilitating *H. pylori* elimination. The idea was to use *M. bovis* BCG encapsulated in chitosan microparticles modified with GlcNAc (G) to increase the adhesion of CHI MPs to gastric mucosa or with Pluronic F 127 (P) to facilitate the release of *M. bovis* BCG cargo in the gut. We used the procedure of inoculating animals with each variant of CHI MPs separately or simultaneously, expecting the synergistic effects in the gastric and gut milieu. Animals that first received CHI MPs were then inoculated with *H. pylori* to mimic early infection (the assessment point 7 days post-inoculation) or chronic infection (the assessment point 28 days post-inoculation) (15, 42). All animals inoculated only with *H. pylori* were successfully infected with these bacteria as confirmed by histological detection of HLO in the gastric tissue or SEM imaging, and by assessment of *cagA* gene copy number. Moreover, FTIR spectra of serum samples of *H. pylori-*infected guinea pigs showed characteristic absorption bands (52). FTIR spectra of sera from *H. pylori*-infected animals after 7 days from inoculation corresponded to increased levels of IgG, CRP and TNF-α, indicating the ongoing inflammatory response and the development of humoral response of the host. Results were positive for animals pre-exposed to CHI MPs loaded with *M. bovis* BCG and then infected with *H. pylori*, after 7 days from the last *H. pylori* dose. However, after 28 days from inoculation of animals with *H. pylori*, the levels of these biomarkers were close to control, which may suggest neutralization of inflammatory response and a clearance of IgG antibodies. It has been shown that IgM and IgG antibodies produced during *H. pylori* infection can form immune complexes with *H. pylori* components, which may support the idea of IgG diminishing in the chronic phase of infection (53–56).

In the gastric tissue of animals receiving first G/P variant of CHI MPs loaded with *M. bovis* BCG and then inoculated with *H. pylori*, the *cagA* qPCR was negative in 4 of 5 animals after 28 days from the last inoculation of them with *H. pylori*. Even in one positive sample, the *cagA* copy number was significantly lower compared to the *cagA* copy number in the gastric tissue of animals 7 days after the last inoculation with *H. pylori*, indicating the ongoing elimination of these bacteria. These data prompted us to search whether the studied CHI MPs alone and/or released *M. bovis* BCG neutralize *H. pylori* binding to gastric mucosa or influence immune mechanisms, unspecific or specific, which might be implicated in *H. pylori* elimination in animals receiving *M. bovis* BCG encapsulated in CHI MPs. It has been shown that free *M. bovis* BCG by reducing gastric MUC5AC reduced *H. pylori* adhesion (27). It may be due to shedding or degradation of mucus related to mycobacterial activity. As shown by Van der Brink et al. (57) on this way the amount of mucosal Lewis determinants utilized by *H. pylori* during adhesion can be diminished. The study by Selwal et al., revealed that mycobacteria modulate mucin synthesis and its properties, influencing the interaction of other microorganisms with mucus (58). It has been shown that *M. bovis* was shed with nasal mucus of cattle infected intranasally or via intratracheal route (59). Mycobacteria produce sulfotransferases and sulfatases, which may remove receptors for *H. pylori* adhesins like heparan sulphate (60, 61).

Regarding the immunomodulatory properties of *M. bovis* BCG, we examined gastric infiltration of macrophages consisting the first line of immune response to infectious agents and implicated in the development of specific acquired immune responses. *H. pylor*i component like LPS negatively modulate macrophage phagocytic activity *in vitro* (24, 28, 48). It was interesting that exposure of macrophages to *M. bovis* BCG *in vitro* resulted in immune training of these cells and upregulation of their phagocytic activity towards fluorescently labelled *E. coli* particles, which was diminished by *H. pylori* LPS (41). In this study, in gastric tissue from animals receiving G/P CHI MPs loaded with *M. bovis* BCG and then infected with *H. pylori*, the number of macrophages was significantly increased compared to animals inoculated only with *H. pylori*, 7 days after the last inoculation, indicating the role of mycobacteria. After 28 days from inoculation with *H. pylori*, the infiltration of macrophages was still enhanced, however, it was lower than 7 days post-inoculation, which may be related to *H. pylori* elimination and reduced demand on these cells. Alternatively, the lower number of macrophages after 28 days could be related to macrophage apoptosis linked to intracellular persistence of mycobacteria (62).

To know whether the increased macrophage infiltration in the gastric tissue of animals inoculated with *H. pylori* or those receiving CHI MPs and then infected with *H. pylori* was not the effect of resident cells responding to chemokine signals, we analyzed the hematopoietic markers CD34, CD117 of myelocytic precursors in the bone marrow cells reflecting proliferation and differentiation of monocytes and macrophages. We also assessed neutrophil Ly6G marker, as neutrophils are involved in the early inflammatory response. In all tested variants, the highest increases in CD34-, CD117, and Ly6G-positive cells were in the bone marrow of animals receiving G/P CHIMPs loaded with *M. bovis* and then infected with *H. pylori*, at 7 or 28 days post-inoculation. This may indicate that *M. bovis* BCG, in conjunction with *H. pylori*, may increase the number of myeloid bone marrow precursors and then their peripheral load and local deposition in the gastric tissue. The study by Fol et al. revealed that in the bone marrow from immunocompetent C57BL/6 mice and C3H mice or mice immunocompromised with cyclophosphamide, vaccinated with wild-type *M. bovis* BCG or with recombinant *M. bovis* BCG secreting murine IL-18 (rBCG/IL-18), there was an increased number of myeloblasts and promyelocytes (63).

It was interesting that *ex vivo* only BMDM from animals inoculated with *H. pylori* alone after 7 and 28 days from inoculation secreted TNF-α the cytokine corresponding to early inflammation, while BMDM from animals exposed only to P/G CHI MPs loaded with *M. bovis* BCG or receiving such CHI MPs and infected with *H. pylori* produced anti-inflammatory IL-10, 28 days from inoculation, which means that the exposure to *M. bovis* BCG may be involved in the maintenance of inflammatory homeostasis. The highest level of chemotactic IL-8 secreted by BMDM from animals receiving CHI MPs loaded with *M. bovis* BCG or such CHI MPs and then infected with *H. pylori*, after 28 days from inoculation compared to animals infected only with *H. pylori* indicate that *M. bovis* BCG may drive the immobilization and activation of lymphocytes characterizing the late phase of inflammatory response. The previous analysis of late cell infiltrate in the gastric tissue from animals infected with *H. pylori* (42, 48) or those, which in these study were exposed to G/P CHI MPs loaded with *M. bovis* BCG and then infected with *H. pylori*, showed enhanced lymphocyte infiltration (unpublished data under consideration).

BMDM from animals receiving G/P CHI MPs loaded with *M. bovis* BCG, or those additionally inoculated with *H. pylori*, showed significantly increased phagocytic activity towards *E. coli* (7 and 28 days post-inoculation) compared to control animals or those inoculated with *H. pylori* alone in association with enhanced CD11d expression. The highest effect was shown after 28 days of inoculation potentially due to macrophage immune training. However, this hypothesis remains not cleared due to similar level of H3K4 methylation in BMDM from animals infected with *H. pylori* alone or those pre-exposed to G/P CHI MPs loaded with *M. bovis* MPs, or those additionally infected with *H. pylori*. In all experimental variants, the formulations used to inoculate animals influenced macrophages at the molecular level. Research in this area must be deepen because previous *in vitro* studies on THP-1-derived macrophages primed with *M. bovis* BCG showed enhanced phagocytic activity compared to cells exposed to *H. pylori* LPS, and this effect was associated with increased total DNA methylation (41). The *in vivo* studies on a model of cattle’s resistant to *M. bovis* vs. susceptible animals indicate that *M. bovis-*infected macrophages exhibit a pro-inflammatory profile with high phagocytic activity (64), which is in agreement with the effects shown in this study.

Given the role of macrophages as antigen-presenting cells, we also asked whether exposing guinea pigs to CHI MPs loaded with *M. bovis* BCG would increase adaptive immune responses. The study by Ancelet et al. showed that oral administration to mice of *M. bovis* BCG in a lipid formulation delivered viable mycobacteria to the mesenteric lymph nodes and conferred protection against an aerosol *M. tuberculosis* challenge (65).

Elimination of *H. pylori* after 28 days of inoculation was observed in animals receiving first G/P CHI MPs loaded with *M. bovis* BCG, suggesting enhanced lymphocyte-dependent immune mechanisms potentially driven by increased activity of macrophages. It has been shown that *M. bovis* BCG, unlike virulent strains of mycobacteria, triggers more extensive apoptosis of infected macrophages as necessary step for eliciting robust protective immunity (62). On the model of alveolar macrophages, the TNF-α signaling or Toll-like receptor 2 triggering of protein kinase-dependent macrophage apoptosis has been suggested (62, 66).

Among the markers of humoral adaptive immunity gastric sIgA level was assed. The exposure of animals to *H. pylori* alone, or only to G/P CHI MPs loaded with *M. bovis* BCG, as well as to CHI MPs and *H. pylori*, induced the production of sIgA after 7 days of inoculation with *H. pylori*. Such antibodies have also been detected after 28 days, however, only in the groups of animals which were exposed only to *H. pylori* or to G/P CHI MPs loaded with *M. bovis* BCG, however the sIgG level was lower than after 7 days. It was interesting that sIgA in the gastric mucosa of animals treated with CHI MPs and then infected with *H. pylori* was similar to that in control animals. sIgA specific to *H. pylori* could be utilized for *H. pylori* recognition and then shed with the mucus. The consequence could be diminished adhesion of *H. pylori* to the gastric tissue. This concept is consistent with earlier results showing reduced MUC5AC in the gastric epithelial cells exposed to *M. bovis* BCG (27).

Association of *H. pylori* infection in *Cavia porcellus* with increased specific anti-*H. pylori* IgG and IgA antibodies indicate the involvement of B and T lymphocytes in driving humoral response and antibody class switching (42, 67). In the spleen of animals exposed to G/P CHI MPs loaded with *M. bovis* BCG and infected with *H. pylori*, after 7 days of inoculation, the number of germinal centers in lymphoid follicles homing activated B lymphocytes was increased. After 28 days, this effect was observed in animals infected with *H. pylori* alone, in those receiving only G/P CHI MPs loaded with *M. bovis* BCG, or in those exposed to such MPs and then infected with *H. pylori*. In the last group, the highest number of germinal centers in the splenic lymphoid follicles indicated a synergistic effect of CHI MPs loaded with *M. bovis* BCG and *H. pylori* on splenocyte activation. This concept has been supported by the increased proliferative activity of splenocytes from animals receiving G/P CHI MPs loaded with *M. bovis* BCG, or those additionally infected with *H. pylori*, when exposed *ex vivo* in cell cultures to *M. bovis* BCG, suggesting that used CHI MPs may be involved in the clonal expansion of lymphocytes. Lasco et al., using the model of guinea pig, showed the increased proliferative activity of splenocytes in response to live attenuated *M. tuberculosis* H37Ra or to heat-killed virulent *M. tuberculosis* H37RV but not to classic *Enterobacterial* LPS upon prior vaccination with *M. bovis* BCG (68).

In our model, the proliferation of splenocytes from animals inoculated only with *H. pylori* was inhibited in the presence of bacterial LPS, and this effect was reversed in cultures of splenocytes from animals receiving CHI MPs loaded with *M. bovis* BCG. Similarly, the reduced phagocytic activity of macrophages in the milieu of *H. pylori* LPS could be reversed by exposing these cells to *M. bovis* BCG, potentially due to immune training (41). In this study, an increased proliferation of splenocytes from animals infected only with *H. pylori*, isolated after 28 days from inoculation, has been observed *in vitro* in response to CagA which can drive cell hyperproliferation and/or cytotoxicity, depending on the cell type and the exposure time (30, 69, 70).

The initiated adaptive immune response could be specific to *H. pylori* or mycobacterial antigens, however, the studied CHI MPs containing chitosan with adjuvant properties could potentially increase the total number of lymphocytes supporting elimination of *H. pylori* infection.

To sum up, pre-exposure of guinea pigs to CHI MPs loaded with *M. bovis* BCG before inoculation with *H. pylori* prevented the development of infection with these bacteria as shown by quantitative *cagA* polymerase chain reaction. This inhibitory effect was associated with propagation of myeloid precursors in the bone marrow, enhanced macrophage infiltration in the gastric tissue and enhanced macrophage phagocytic activity, which in animals inoculated only with *H. pylori* was negatively modulated. The protective effect of CHI MPs with *M. bovis* BCG was also associated with increased gastric concentrations of secretory IgA, activation of splenic germinal centers and enhanced splenocyte proliferation. The type of CHI MPs modification may influence the above effects, as shown by using P or G CHI MPs variants alone or in combination. The obtained results indicate the immunomodulatory properties of CHI MPs loaded with *M. bovis* BCG improving innate and adaptive immune mechanisms, facilitating control of *H. pylori* infection in the guinea pig model. The biocompatibility of CHI MPs loaded with *M. bovis* BCG used in this study has been confirmed and potential cytotoxic effects in different organs, including the liver, have been excluded. However, in the context of *H. pylori* infection, an increased permeability of the gastric and intestinal epithelium caused by soluble components of these bacteria should be carefully considered in further studies and data interpretation, as the condition of these barriers may influence the effects induced by mycobacteria.

## 5 Conclusions

**Figure 10:**
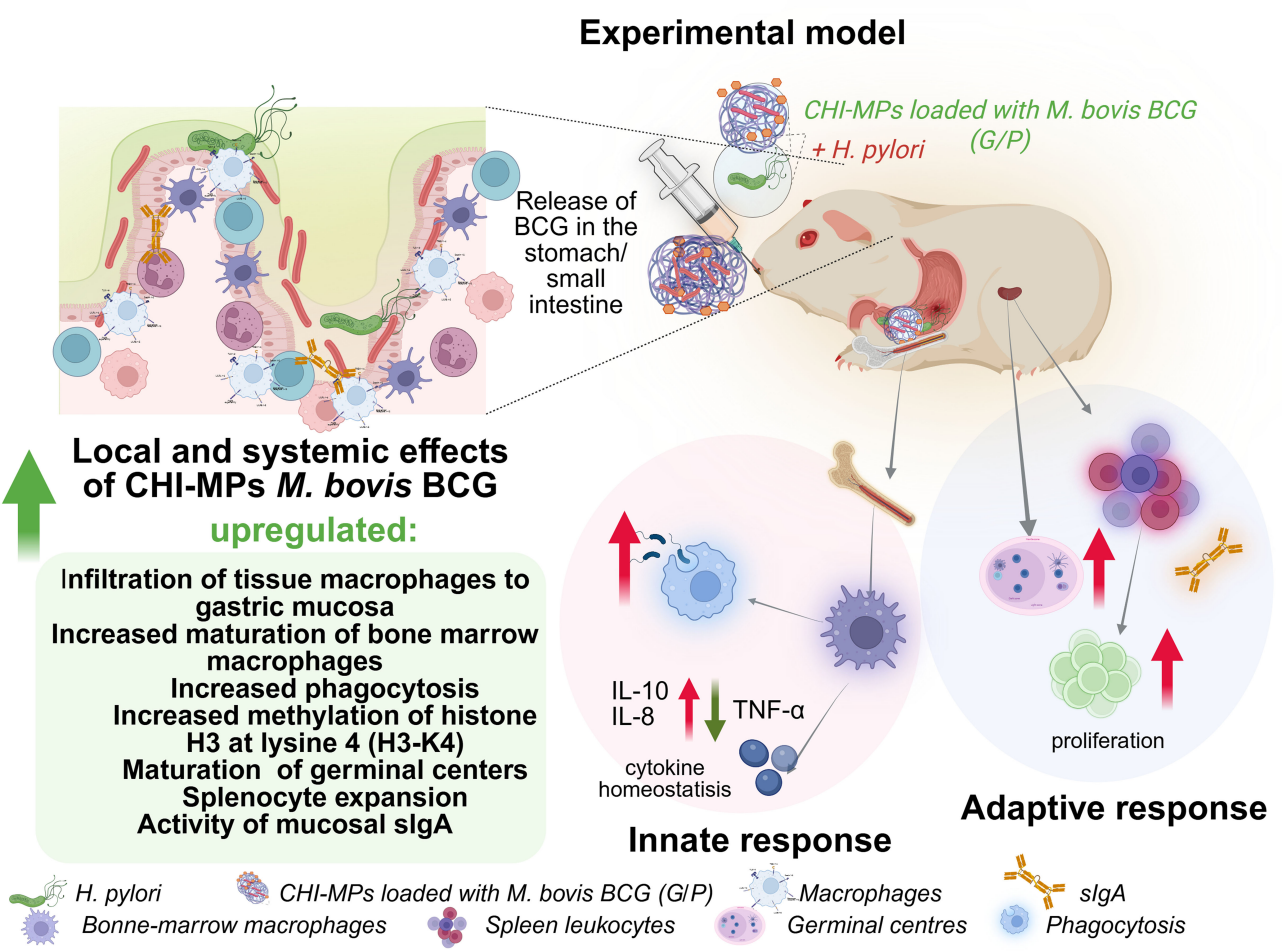
Immunomodulatory potential of MPs loaded with *M. bovis* BCG during *H. pylori* infection. Created in BioRender. Mikolajczyk-Chmiela, M (2026). https://BioRender.com/5gti4mh.

This initial *in vivo* study using the model of *Cavia porcellus* demonstrates that modified CHI MPs loaded with live *M. bovis* BCG, administered orally before inoculating the animals with *H. pylori*, trigger innate and adaptive immune responses that help prevent or reduce *H. pylori* colonization due to CHI MPs immunomodulatory properties (**Figure 10**). This inhibitory effect was associated with propagation of myeloid precursors in the bone marrow, enhanced macrophage infiltration in the gastric tissue and enhanced macrophage phagocytic activity, which in animals inoculated only with *H. pylori* was negatively modulated. The protective effect of CHI MPs with *M. bovis* BCG was also associated with increased gastric concentration of secretory IgA, activation of splenic germinal centers and enhanced splenocyte proliferation. In the future, CHI MPs loaded with live *M. bovis* BCG may serve as a prototype vaccine protecting against *H. pylori* infection in humans. The guinea pig stomach anatomically and physiologically resembles the human stomach. A similar inflammatory response onset has also been observed in guinea pigs, and guinea pig gastric epithelial cells express a homolog of the human proinflammatory cytokine IL-8 with chemotactic activity. Guinea pigs also express the complement system. Like humans, guinea pigs require a diet rich in vitamin C. Further research using a larger number of animals per group is necessary to strengthen the results obtained in these study, indicating the immunomodulatory activity of *M. bovis* BCG delivered by CHI MPs to gastrointestinal tract of guinea pigs resulting in inhibition of *H. pylori* infection.

## Data Availability

This published article includes all data generated or analyzed during this study. The representative raw data is posted at the link: http://hdl.handle.net/11089/56990.
