# Supplementary material for: Upregulation of innate and adaptive immune mechanisms facilitating prevention of gastric Helicobacter pylori infection in guinea pigs by per os administration of chitosan microparticles loaded with Mycobacterium bovis BCG
Source: Front Immunol. 2026 Mar 18;17:1771052. doi: 10.3389/fimmu.2026.1771052 (PMC13038921; doi:10.3389/fimmu.2026.1771052)
Supplement: Supplementary file 1 [file DataSheet1.docx]

Supplementary Material

**Upregulation of innate and adaptive immune mechanisms facilitating prevention of gastric *Helicobacter pylori* infection in guinea pigs by *per os* administration of chitosan microparticles loaded with *Mycobacterium bovis* BCG.**

Weronika Gonciarz*^1^, Marek Brzeziński^2^, Agnieszka Wosiak^3,4,^ Agnieszka Jeleń^3,4,^ , Ewa Balcerczak^3,4,^ , Magdalena Chmiela^1*^


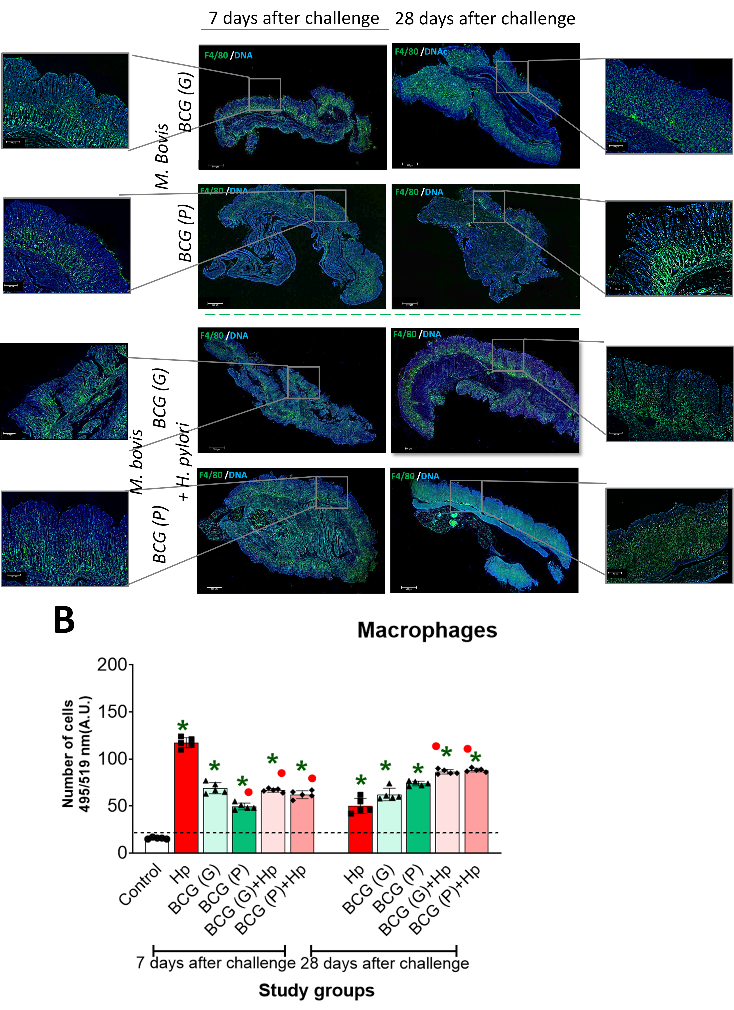


**Supplementary Figure 1. Gastric tissue macrophage infiltration.**

(A) Representative images of macrophage infiltration (green) in gastric tissue specimens of guinea pigs under the study. Macrophages were stained with primary mouse anti-F4/80 antibodies and then with antibodies towards mouse immunoglobulins fluorescently labeled AlexaFluor 488. Cell nuclei were stained with 4',6-Diamidino-2-phenylindole dihydrochloride (DAPI). The images were observed under a confocal microscope at appropriate wavelengths: for DAPI, 345 nm (excitation) and 455 nm (emission); for AlexaFluor 488, 490 nm (excitation) and 525 nm (emission), magnification 10x or 40x water immersion (Microhub Leica MICA). Animals revised: only *H. pylori*, only chitosan microparticles (CHI MPs) loaded with *Mycobacterium bovis* BCG or CHI MPs and then were inoculated with *H. pylori*. *M. bovis* BCG (G/P) - chitosan microparticles loaded with *M. bovis* BCG modified with GlcNAc (G) or with Pluronic F127 (P). (B) The number of macrophages was assessed by analyzing the tissue area 1 cm x 1 cm; 5 fields from each tissue. Macrophage assessment scale: grade 1 – single macrophages in the imaged area (grade 1 - number of cells up to 15; grade 2 – several macrophages mainly below the muscularis mucosa (number of cells up to 50; grade 3 – several macrophages below and above the muscularis mucosa (number of cells up to 75); grade 4 – infiltration of macrophages through whole tissue (number of cells above to 100). Results are presented as the number of macrophages (*arbitrary units* A.U. ) ± range of three independent experiments. Statistical significance for p <0.05 in the non-parametric Mann-Whitney or Kruskal-Wallis U test. * Animals non-treated (control groups) vs. treated with chitosan microparticles (CHI MPs) loaded with *M. bovis* BCG or inoculated with *H. pylori* or first receiving such CHI MPs and then inoculated with *H. pylori*. ● Animals treated with *H. pylori* vs. animals treated with CHI MPs loaded with *M. bovis* BCG or first receiving CHI MPs loaded with *M. bovis* BCG and then inoculated with *H. pylori* (comparison by treatment times). Animals received: only *H. pylori*, only CHI MPs loaded with *M. bovis* BCG modified with N-acetylglucosamine (GlcNAc) (G) or with Pulonic F127 (P) or such CHI MPs and then were inoculated with *H. pylori*.


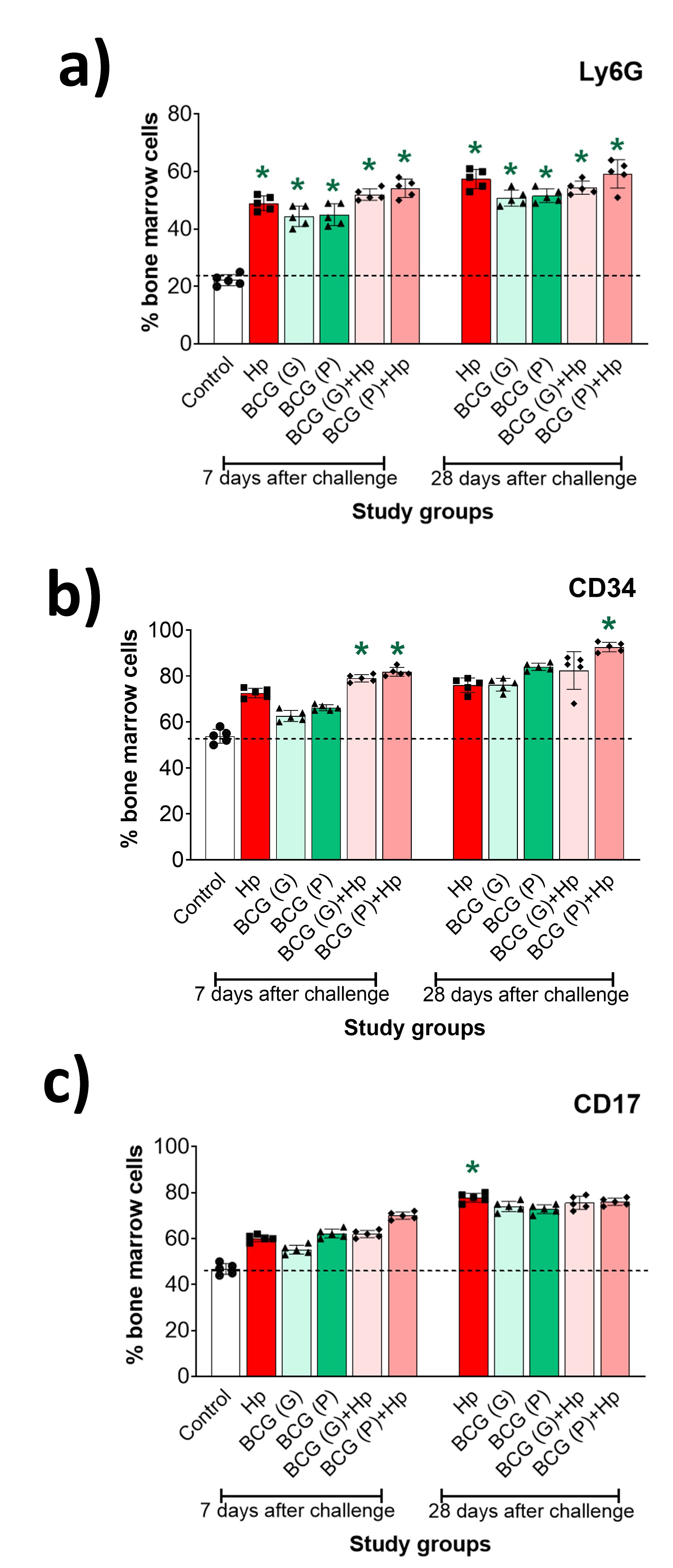


**Supplementary Figure 2. Phenotyping of bone marrow cells.**

Cells were stained with primary rabbit anti-Ly6G antibodies (a), anti-CD34 (b) or anti-CD117 (c) antibodies and then with secondary antibodies fluorescently labelled AlexaFluor 488 (green) or AlexaFluor 568 (red). Representative images were prepared under a fluorescence microscope (Zeiss, Axio Scope, A1, Oberkochen, Germany) using 20x/0.32 objective at the appropriate wavelengths for DAPI (358 nm excitation, 461 nm emission), AlexaFluor488 (490 nm excitation, 525 nm emission), and AlexaFluor 647 (650nm excitation, 671nm emission). Percentage (%) of CD34, CD117, or LY6G-positive cells vs. nucleated cells. Statistical significance for p <0.05 in the non-parametric Mann-Whitney or Kruskal-Wallis U test. * Animals non-treated (control groups) vs. animals treated with CHI MPs loaded with *M. bovis* BCG alone or inoculated only with *H. pylori*, or first receiving CHI MPs loaded with *M. bovis* BCG and then inoculated with *H. pylori*. ● animals inoculated with *H. pylori* vs. animals treated with CHI MPs loaded with *M. bovis* BCG or first receiving CHI MPs loaded with *M. bovis* BCG and then inoculated with *H. pylori* (comparison concerning treatment time). Animals received: only *H. pylori*, only chitosan microparticles (CHI MPs) loaded with *M. bovis* BCG or such CHI MPs and then were inoculated with *H. pylori.* BCG (G) or BCG (P) - CHI MPs loaded with *M. bovis*-BCG modified with N-acetylglucosamine (GlcNAc) or with Pulronic F127 (P), respectively. Statistical significance for p <0.05 in the non-parametric Mann-Whitney or Kruskal-Wallis U test. * Animals non-treated (control groups) vs. animals treated with CHI MPs loaded with *M. bovis* BCG alone or inoculated only with *H. pylori*, or first receiving CHI MPs loaded with *M. bovis* BCG and then inoculated with *H. pylori*. ● animals inoculated with *H. pylori* vs. animals treated with CHI MPs loaded with *M. bovis* BCG or first receiving CHI MPs loaded with *M. bovis* BCG and then inoculated with *H. pylori* (comparison concerning treatment time).


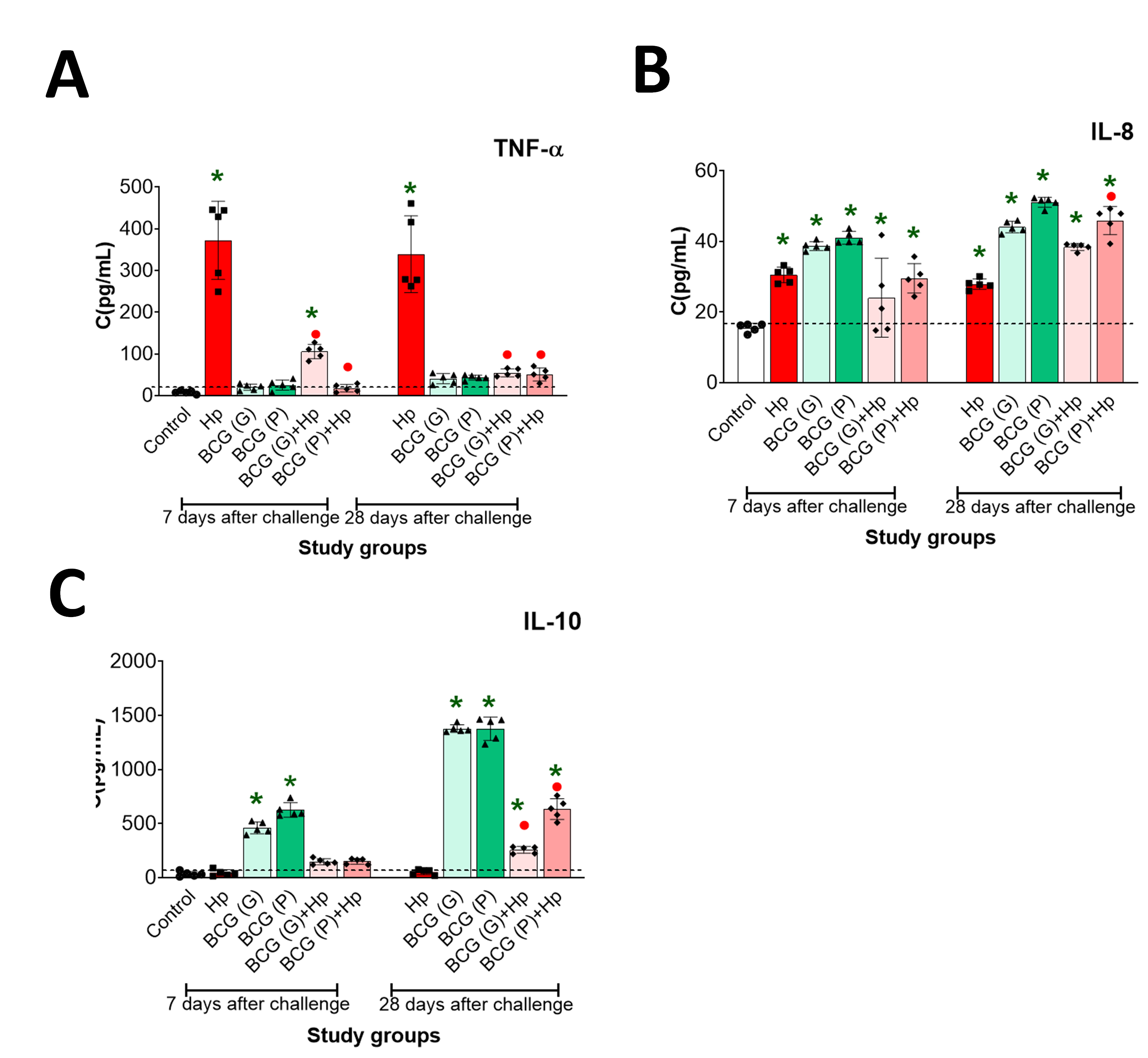


**Supplementary Figure 3. BMDM cytokine profile.**

The level of TNF-a (A), IL-8 (B) and IL-10 (C) was estimated in ELISA assay (sensitivity 1 pg/mL). Results are presented as mean ± range of three independent experiments. Statistical significance for p <0.05 in the non-parametric Mann-Whitney or Kruskal-Wallis U test. * Animals non-treated (control group) vs. animals treated with chitosan microparticles (CHI MPs) loaded with *M. bovis* BCG or with *H. pylori,* or first with CHI MPs and then infected with *H. pylori*. ● animals treated with *H. pylori* vs. animals treated with CHI MPs loaded with *M. bovis*BCG or first receiving CHI MPs loaded with *M. bovis* BCG and then infected with *H. pylori* (comparison by treatment times). Animals revised: only *H. pylori*, only chitosan microparticles (CHI MPs) loaded with *M. bovis* BCF, modified with N-acetylglucosamine (GlcNAc) (G) or with Pluronic F 127 (P) or first received such CHI MPs and then were infected with *H. pylori*.


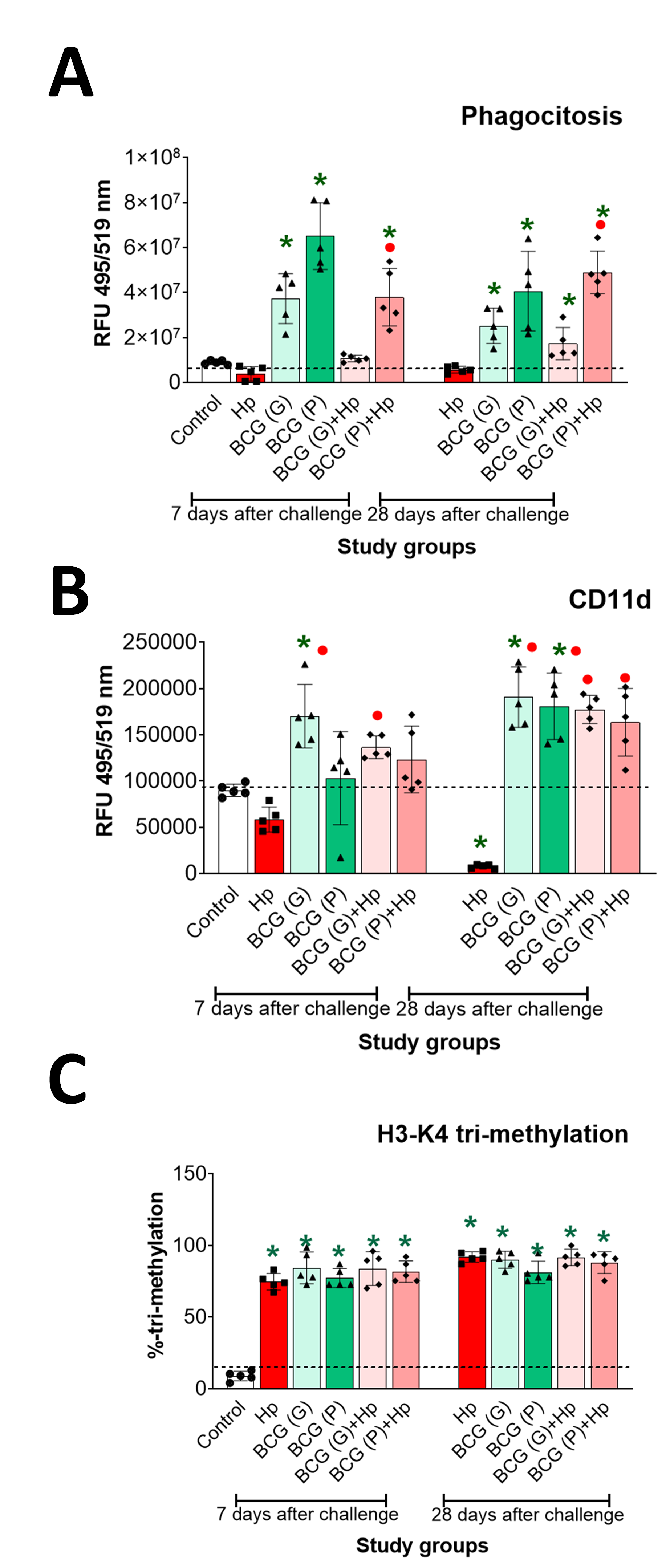


**Supplementary Figure 4 BMDM phagocytic activity and H3-K4 methylation.**

(A) - The phagocytic activity of guinea pig bone marrow-derived macrophages (BMDM) towards fluorescently labelled *E. coli* (Vybrant phagocytosis assay kit). (B) - CD11d expression on BMDM. (C) - Methylation of histone H3 at lysine 4 (H3-K4). Phagocytosis of *E. coli* fluorescently labeled particles by BMDM was measured using a multifunctional reader, SpectraMax i3 (Molecular Devices, San Jose, CA, USA), at an excitation wavelength of 495 nm and an emission wavelength of 515 nm. CD11d deposition was assessed by staining cells with primary mouse anti-CD11d antibodies and then with secondary antibodies fluorescently labeled AlexaFluor 488 (green) (490 nm excitation, 525 nm emission), AlexaFluor 568 (591 nm excitation, 608 nm emission). Methylation of H3-K4 was assed calorimetrically and shown as percentage of H3-K4 positive cells. Results (A, B, C) are presented as ratio of median fluorescence units (RFU) ± range of three independent experiments. Statistical significance for p <0.05 in the non-parametric Mann-Whitney or Kruskal-Wallis U test. * Animals non-treated (control groups) vs. treated with chitosan microparticles (CHI MPs) loaded with *M. bovis* BCG or inoculated with *H. pylori* or first receiving such CHI MPs and then inoculated with *H. pylori*. ● Animals treated with *H. pylori* vs. animals treated with CHI MPs loaded with *M. bovis* BCG or first receiving CHI MPs loaded with *M. bovis* BCG and then inoculated with *H. pylori* (comparison by treatment times). Animals received: only *H. pylori*, only CHIMPs loaded with *M. bovis* BCG modified with N-acetylglucosamine (GlcNAc) (G) or with Pulonic F127 (P) or such MPs and then were inoculated with *H. pylori*.


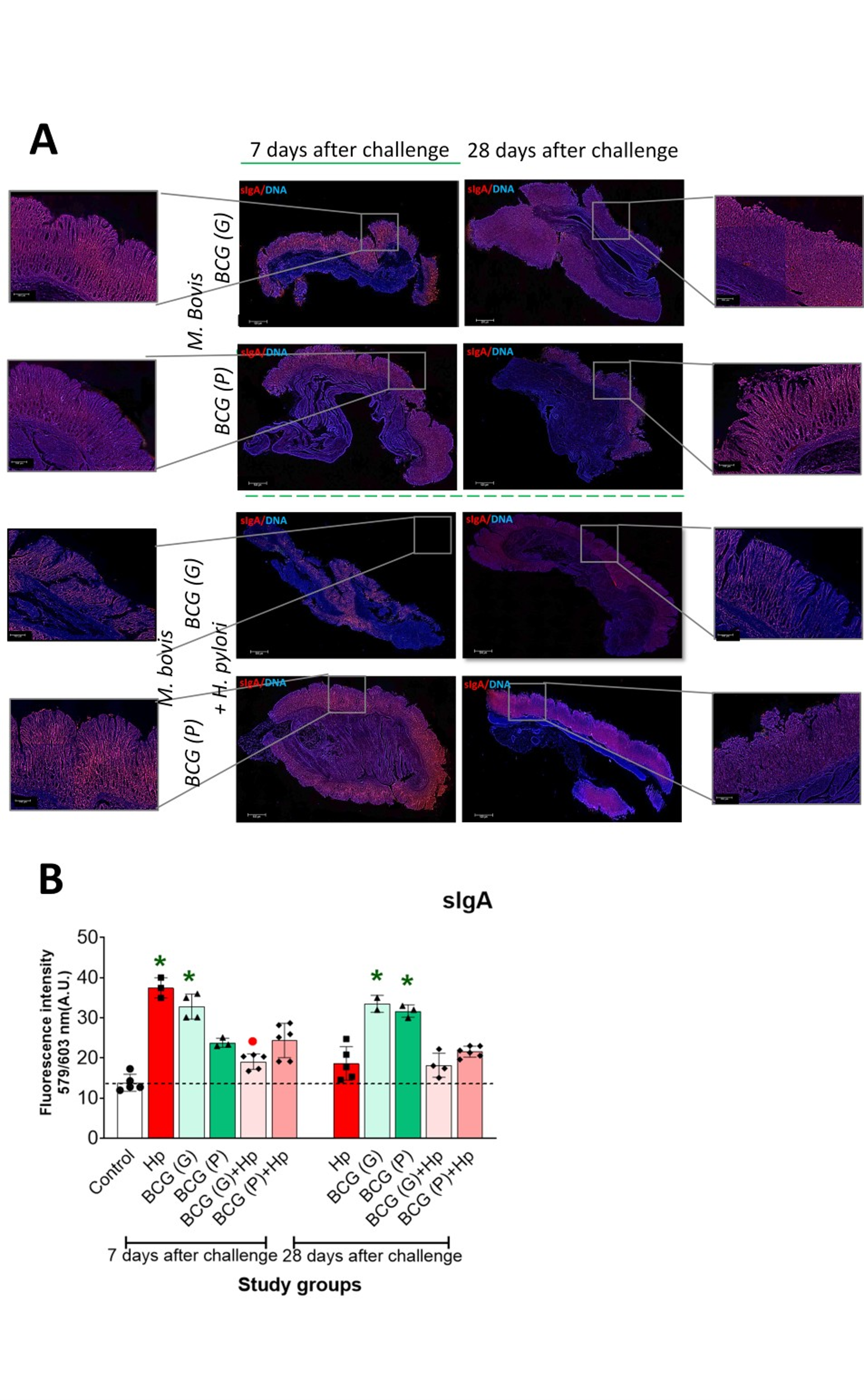


**Supplementary Figure 5. Gastric tissue sIgA.**

(A) - Representative images of IgA secretion (red) in gastric tissue specimens of the guinea pig were assessed by staining with primary rabbit anti-IgA antibodies and then secondary antibodies, fluorescently labelled AlexaFluor 647. Representative images were prepared under a confocal microscope at appropriate wavelengths: DAPI, 345 (excitation) and 455 (emission); AlexaFluor 647, 650 nm (excitation) and 671 nm (emission), magnification 10x or 40x water immersion (Microhub Leica MICA). (B) The level of sIgA secretion was assessed by analyzing the tissue area 1 cm x 1 cm; 5 fields from each tissue. sIgA assessment scale shown in fluorescence units (FU, 1- up to 15 FU, 2- up to 25 FU, 3- up to 35 FU, 4-above 35 FU. A.U (arbitrary units; corresponding relative fluorescence intensity). Results are presented as relative fluorescence intensity (*arbitrary units* A.U. ) ± range of three independent experiments. Statistical significance for p <0.05 in the non-parametric Mann-Whitney or Kruskal-Wallis U test. * Animals non-treated (control groups) vs. treated with chitosan microparticles (CHI MPs) loaded with *M. bovis* BCG or inoculated with *H. pylori* or first receiving such CHI MPs and then inoculated with *H. pylori*. ● Animals treated with *H. pylori* vs. animals treated with CHI MPs loaded with *M. bovis* BCG or first receiving CHI MPs loaded with *M. bovis* BCG and then inoculated with *H. pylori* (comparison by treatment times). Animals revised: only *H. pylori*, only BCG (G) or BCG (P) - chitosan microparticles loaded with *Mycobacterium bovis*-BCG modified with N-acetylglucosamine (GlcNAc) (G) or CHI MPs loaded with *Mycobacterium bovis*-BCG modified with Pulonic F127 (P), respectively or such CHI MPs and then were infected with *H. pylori*


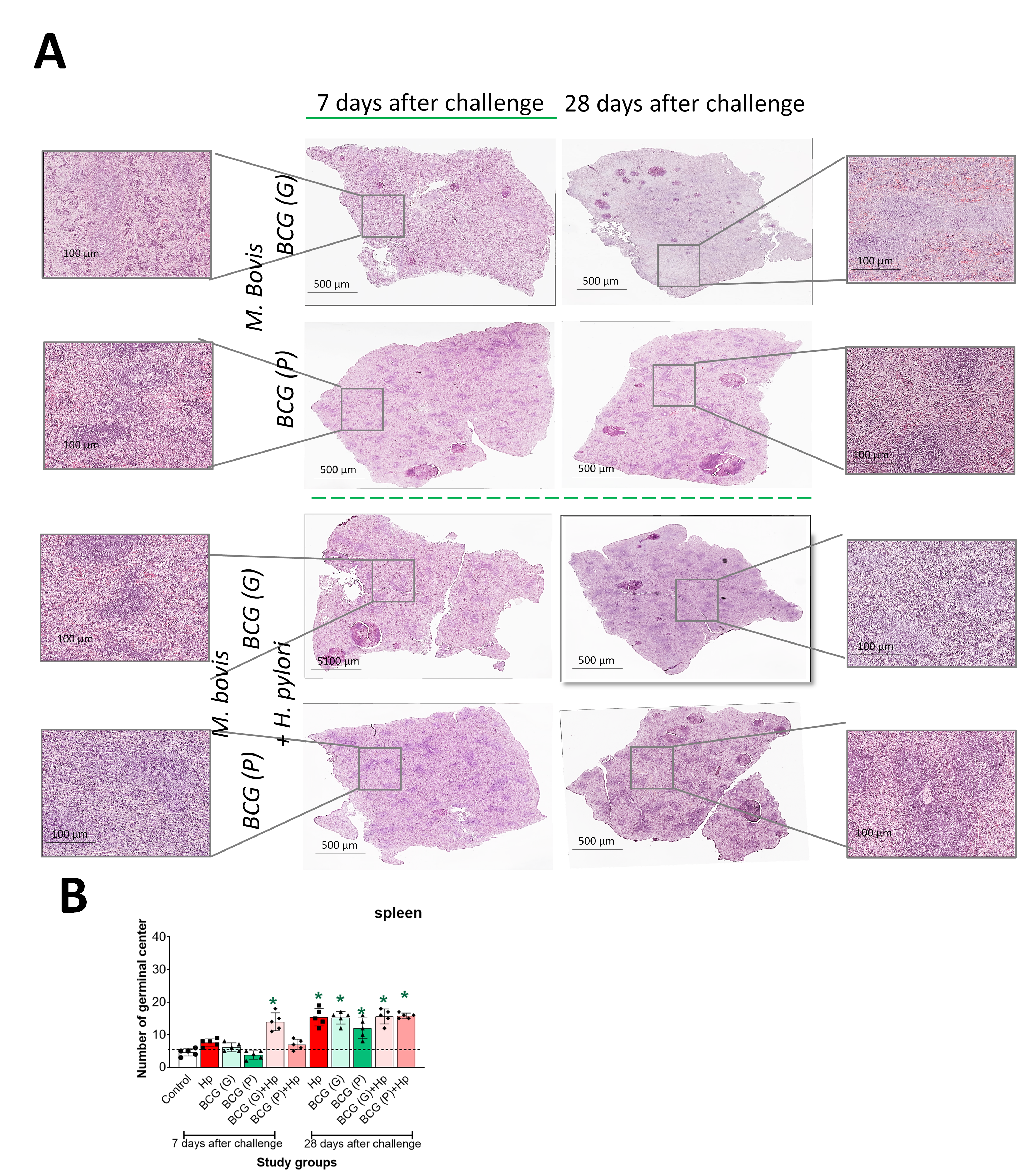


**Figure 6 Propagation of splenic germinal centers.**

(A) - Representative images of spleen specimens of studied guinea pigs stained with H&E from a confocal microscope in transmitted light, magnification 10x, or 40x water immersion (Microhub Leica MICA). (B) - percentage of germinal centers in lymphoid follicles in the spleen sections. Results are presented as mean ± range of three independent experiments. In each section, 3 fields (5 cm x 5 cm) were assessed. Statistical significance for p <0.05 in the non-parametric Mann-Whitney or Kruskal-Wallis U test. * Animals non-treated (control groups) vs. treated with chitosan microparticles (CHI MPs) loaded with *M. bovis* BCG or inoculated with *H. pylori* or first receiving such CHI MPs and then inoculated with *H. pylori*. ● Animals treated with *H. pylori* vs. animals treated with CHI MPs loaded with *M. bovis* BCG or first receiving CHI MPs loaded with *M. bovis* BCG and then inoculated with *H. pylori* (comparison by treatment times). Animals revised: only *H. pylori*, only BCG (G) or BCG (P) - CHI MPs loaded with *Mycobacterium bovis*-BCG modified with N-acetylglucosamine (GlcNAc) (G) or CHI MPs loaded with *Mycobacterium bovis*-BCG modified with Pulonic F127 (P), respectively or such CHI MPs and then were infected with *H. pylori*


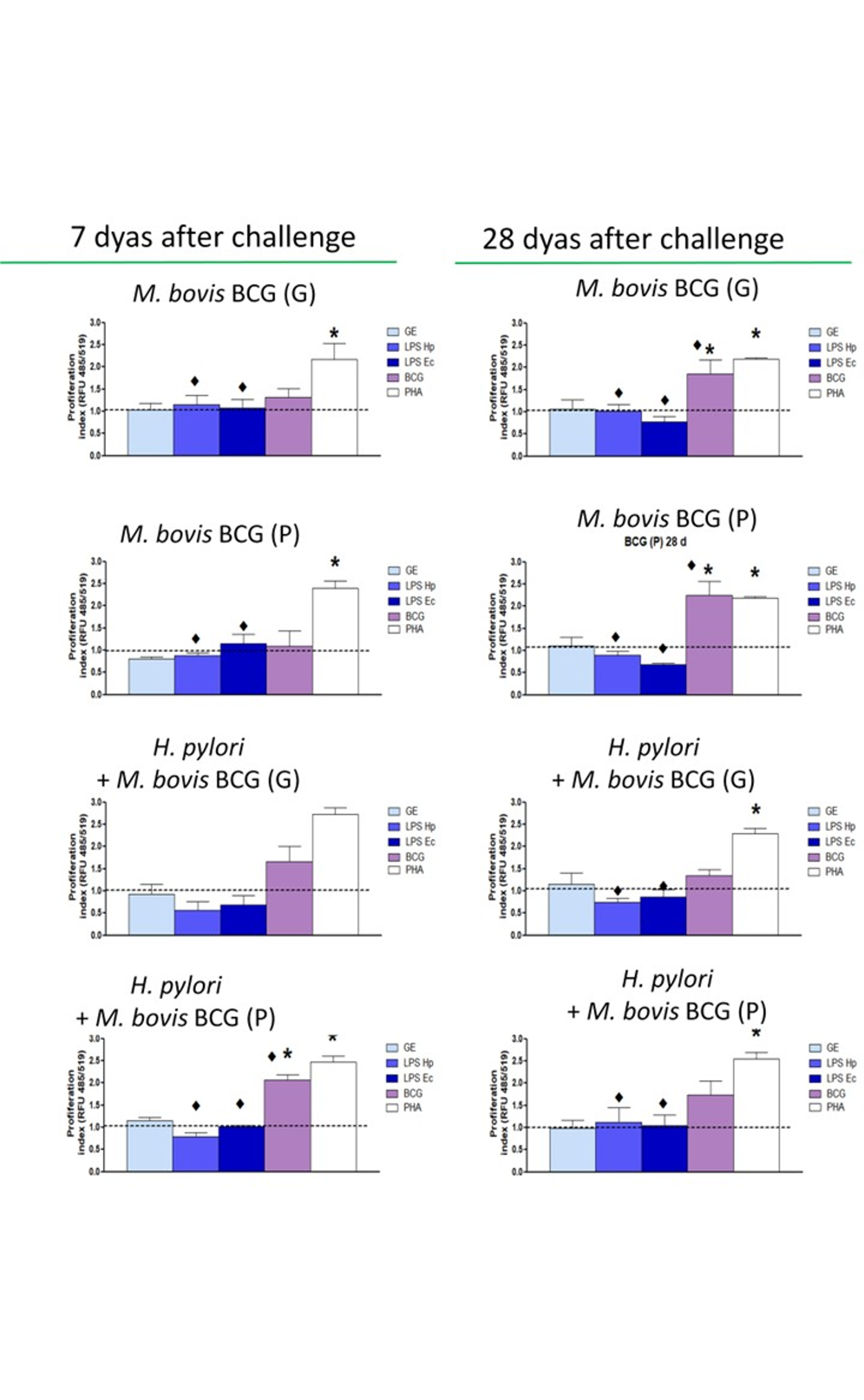
**Supplementary Figure 7. Splenocyte proliferation.**

For the proliferation assay, the following spleen leukocytes were used: untreated or treated for 24 h *in vitro* with *M. bovis* BCG (MOI 1:10), *H. pylori* glycine extracts - GE (10 μg/mL), *H. pylori* lipopolysaccharide - LPS (25 ng/mL) or *E. coli* LPS (25 ng/mL), or phytohemagglutinin - PHA (2 µg/mL). Proliferation was determined using the Invitrogen™ CyQUANT™ Cell Proliferation Assay and reported as the number of cells in culture. Results are presented as mean ± range of three independent experiments. Statistical significance for p <0.05 in the non-parametric Mann-Whitney or Kruskal-Wallis U test. * Animals non-treated (control group) vs. treated with chitosan microparticles (CHI MPs) loaded with *M. bovis* BCG or *H. pylori,* or first receiving CHI MPs loaded with *M. bovis* BCG and then infected with *H. pylori*. ● animals treated with *H. pylori* vs. treated with CHI MPs or *H. pylori*, or first receiving CHI MPs then infected with *H. pylori* (comparison by treatment times). Animals received: only *H. pylori*, only chitosan microparticles (CHI MPs) loaded with *Mycobacterium bovis* modified with N-acetylglucosamine (GlcNAc) (G), or CHI MPs loaded with *M. bovis*-BCG modified with Pluronic F127 (P) or first MPs and then were infected with *H. pylori*.
